# Supplementary material for: Synthesis, Characterization and Catalytic Property Studies for Isoprene Polymerization of Iron Complexes Bearing Unionized Pyridine-Oxime Ligands
Source: Polymers (Basel). 2022 Sep 1;14(17):3612. doi: 10.3390/polym14173612 (PMC9459928; doi:10.3390/polym14173612)
Supplement: Supplementary file 1 [file polymers-14-03612-s001.zip › polymers-1840496-supplementary.pdf]

Supplementary Information

Synthesis, Characterization and Catalytic Property Studies for Isoprene Polymerization of Iron Complexes Bearing Unionized Pyridine-Oxime Ligands

X-ray data for complex Fe2 2

FTIR data for ligands L1–L4 and complexes Fe1–Fe4 5

Mössbauer spectrum of complex Fe1 6

NMR data for ligands and polyisoprene 7

GPC data for polyisoprene 38

Comparison of previous works and this work 45

X-ray data for complex Fe2

Table S1. Crystal data and structure refinement for complex **Fe2**.

Identification Code	211223c
Empirical formula	$C_{14}H_{16}Cl_2FeN_4O_2$
Formula weight	399.06
Temperature	298(2) K
Wavelength	0.71073 A
Crystal system, space group	Orthorhombic, P2(1)2(1)2
	a = 17.0813(18) A alpha = 90 deg.
Unit cell dimensions	b = 7.8822(9) A beta = 117.610(5) deg.
	c = 14.9716(16) A gamma = 90 deg.
Volume	1786.2(3) A^3
Z, Calculated density	4, 1.484 Mg/m^3
Absorption coefficient	1.156 mm^-1
F(000)	816
Crystal size	0.23 × 0.08 × 0.04 mm
Theta range for data collection	2.69 to 25.01 deg.
Limiting indices	-19<=h<=20, -9<=k<=9, -16<=l<=17
Reflections collected / unique	4199/1575 [R(int) = 0.1285]
Completeness to theta = 25.01	99.6 %
Absorption correction	Semi-empirical from equivalents
Max. and min. transmission	0.9552 and 0.7770
Refinement method	Full-matrix least-squares on F^2
Data / restraints / parameters	1575 / 0 / 106
Goodness-of-fit on F^2	1.067
Final R indices [I>2sigma(I)]	R1 = 0.0755, wR2 = 0.2114
R indices (all data)	R1 = 0.0981, wR2 = 0.2289
Largest diff. peak and hole	0.564 and -979 e.A^-3

Table S2. Bond lengths for complex **Fe2**

bond lengths (Å)			
Fe(1)-N(2)#	2.159(4)	C(2)-C(1)	1.476(8)
Fe(1)-N(2)	2.159(4)	C(1)-H(1A)	0.93
Fe(1)-N(1)#	2.283(5)	C(5)-C(4)	1.382(9)
Fe(1)-N(1)	2.283(5)	C(5)-C(6)	1.396(8)
Fe(1)-Cl(1)	2.4871(16)	C(5)-H(5)	0.93
Fe(1)-Cl(1)#	2.4871(16)	C(3)-C(4)	1.402(8)
O(1)-N(2)	1.397(5)	C(3)-H(3)	0.93
O(1)-H(1)	0.82	C(7)-C(6)	1.518(8)
N(2)-C(1)	1.278(7)	C(7)-H(7A)	0.96
N(1)-C(6)	1.345(7)	C(7)-H(7B)	0.96
N(1)-C(2)	1.383(6)	C(7)-H(7C)	0.96
C(2)-C(3)	1.374(7)	C(4)-H(4)	0.93

Table S3. Selected bond angles for complex **Fe3**.

**selected bond angles (°)**			
N(2)#-Fe(1)-N(2)	175.2(2)	N(1)-Fe(1)-Cl(1)#	157.41(11)
N(2)#-Fe(1)-N(1)#	73.66(16)	Cl(1)-Fe(1)-Cl(1)#	99.20(9)
N(2)-Fe(1)-N(1)#	109.70(16)	N(2)-O(1)-H(1)	109.5
N(2)#-Fe(1)-N(1)	109.70(16)	C(1)-N(2)-O(1)	115.3(4)
N(2)-Fe(1)-N(1)	73.66(16)	C(1)-N(2)-Fe(1)	119.1(4)
N(1)#-Fe(1)-N(1)	96.2(2)	O(1)-N(2)-Fe(1)	125.2(3)
N(2)#-Fe(1)-Cl(1)	84.24(12)	C(6)-N(1)-C(2)	117.6(5)
N(2)-Fe(1)-Cl(1)	92.65(13)	C(6)-N(1)-Fe(1)	128.9(3)
N(1)#-Fe(1)-Cl(1)	157.41(11)	C(2)-N(1)-Fe(1)	111.3(3)
N(1)-Fe(1)-Cl(1)	86.71(11)	C(3)-C(2)-N(1)	122.9(5)
N(2)#-Fe(1)-Cl(1)#	92.65(13)	N(1)-C(2)-C(1)	115.3(5)
N(2)-Fe(1)-Cl(1)#	84.24(12)	N(2)-C(1)-C(2)	117.5(5)
N(1)#-Fe(1)-Cl(1)#	86.71(11)	N(2)-C(1)-H(1A)	121.3

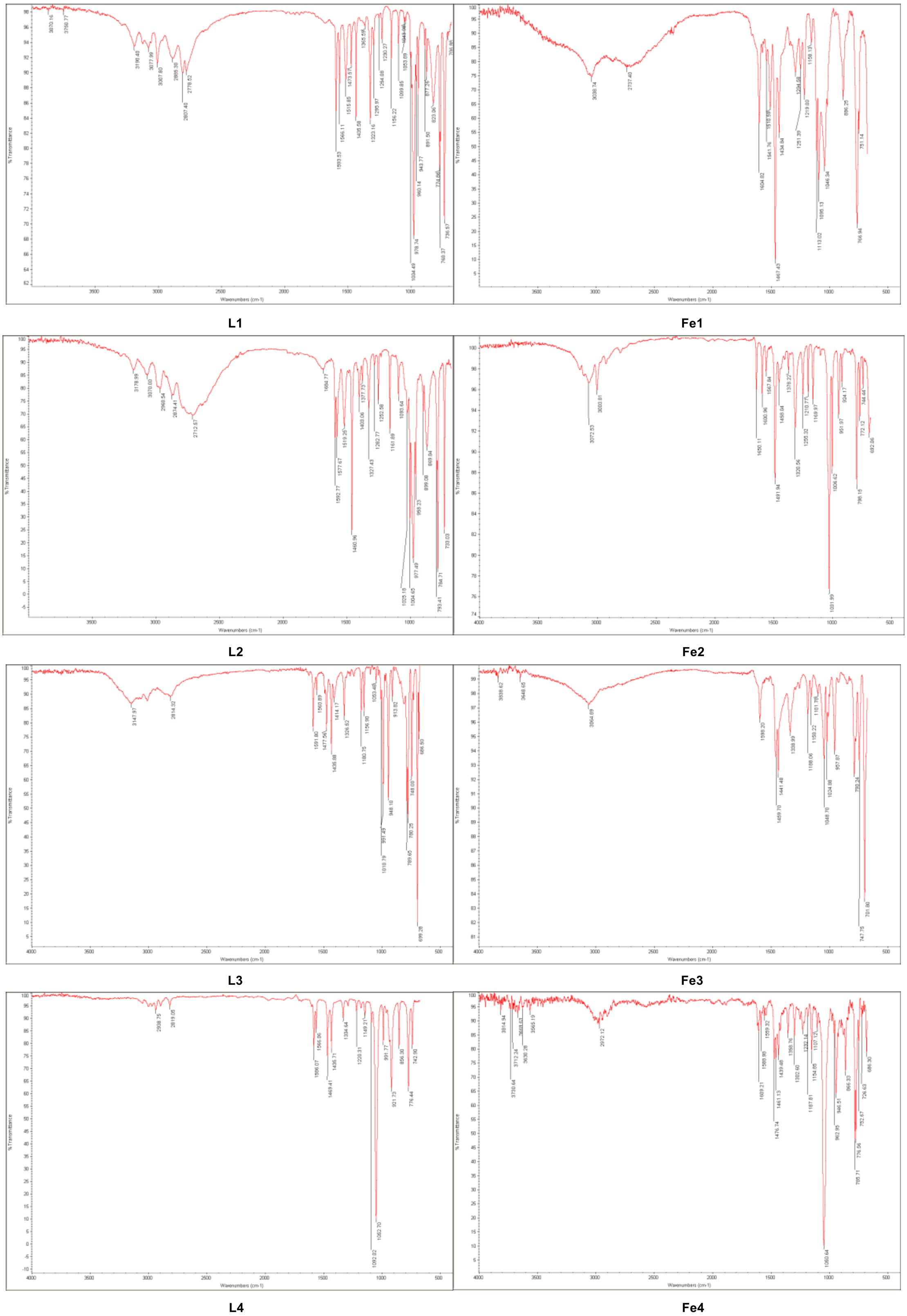

Figure S1. FTIR data for ligands **L1–L4** and complexes **Fe1–Fe4**

Ch 512	Mode: Area	χ^2=801.73 (1.616)
**Parameter**	**Value**	**StD**
(0) Base Line:	195451.86492	22.3
**Doublet (1):**	**[60.50%]**	**[Quadrupole]**
(1) AMPLITUDE:	19035.63836	136
(1) ISOMER SHIFT:	0.32630	0.00161
(1) Q. SPLITTING:	0.39159	0.00225
(1) LINE WIDTH:	0.38247	0.00416
**Doublet (Fe2+):**	**[25.95%]**	**[Quadrupole]**
(2) AMPLITUDE:	8164.73761	205
(2) ISOMER SHIFT:	1.32868	0.00585
(2) Q. SPLITTING:	2.94218	0.0119
(2) LINE WIDTH:	0.58773	0.0178
**Doublet (2):**	**[13.55%]**	**[Quadrupole]**
(3) AMPLITUDE:	4261.63844	149
(3) ISOMER SHIFT:	1.32473	0.00535
(3) Q. SPLITTING:	1.05206	0.0105
(3) LINE WIDTH:	0.36546	0.0144
**Total spectral area:**	62924.02881	

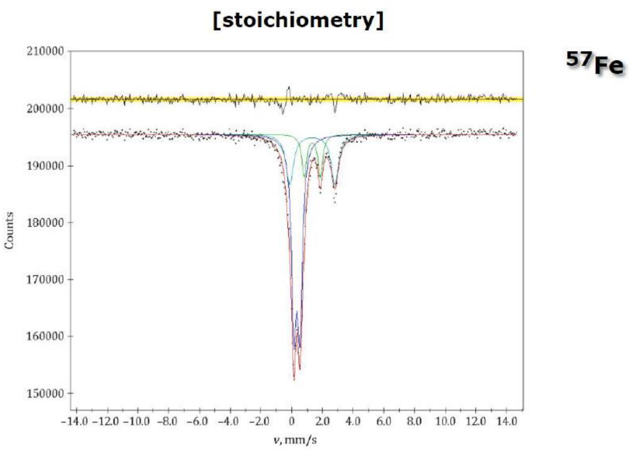

Figure S2. Mössbauer spectrum of complex **Fe1**

NMR data for ligands and polyisoprene.

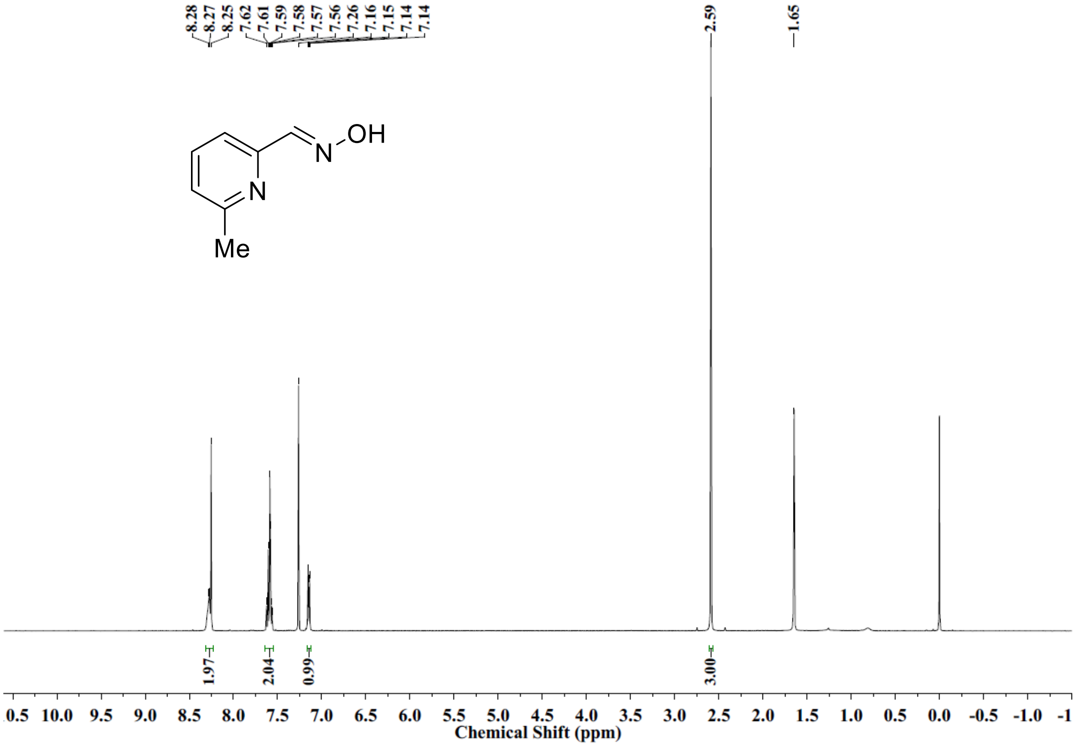

^{1}H NMR of **L2**.

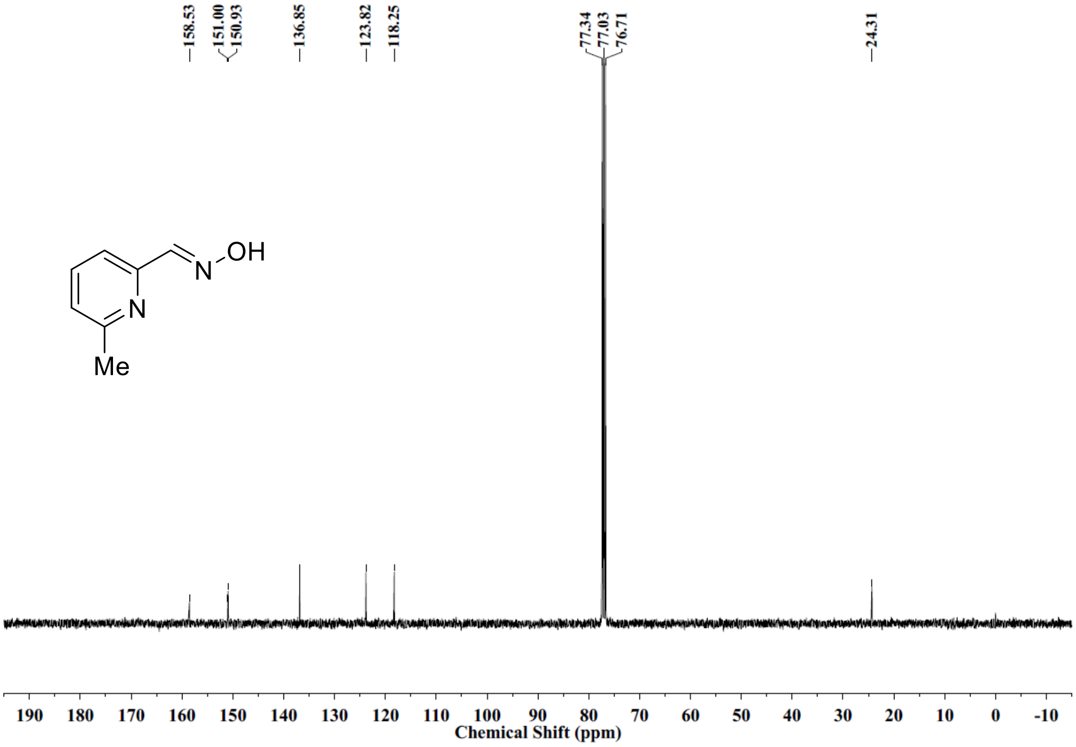

^{13}C NMR of **L2**.

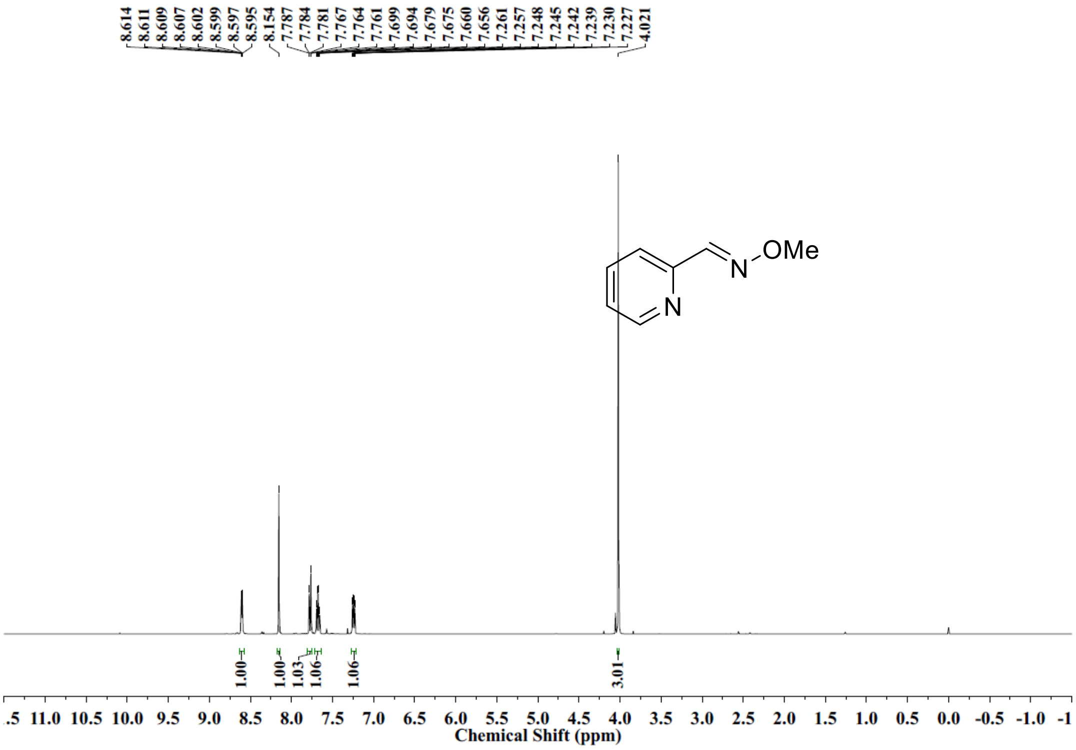

^{1}H NMR of **L4**.

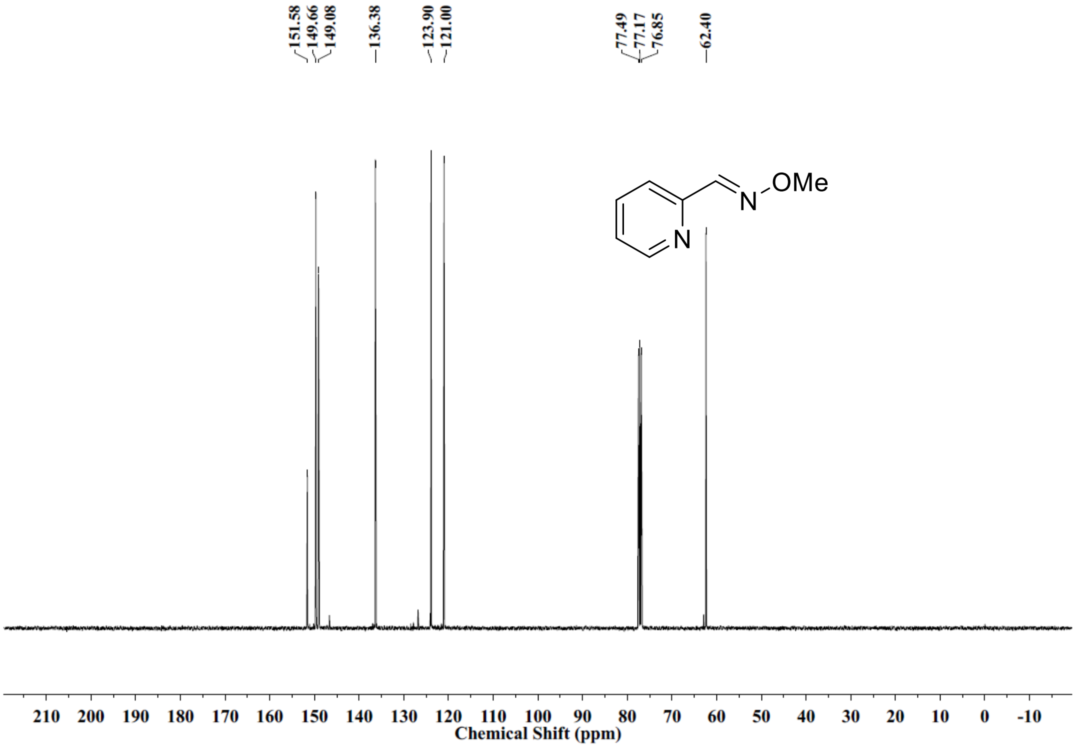

^{13}C NMR of **L4**.

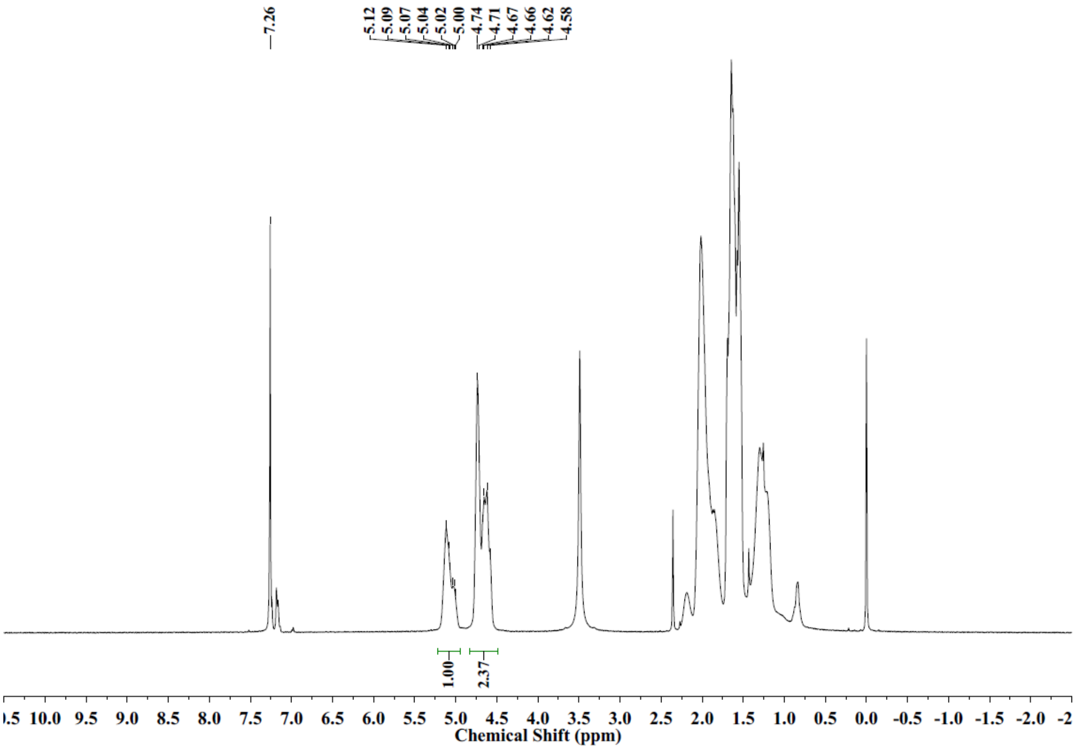

Table 1. entry 1 (^{1}H MMR).

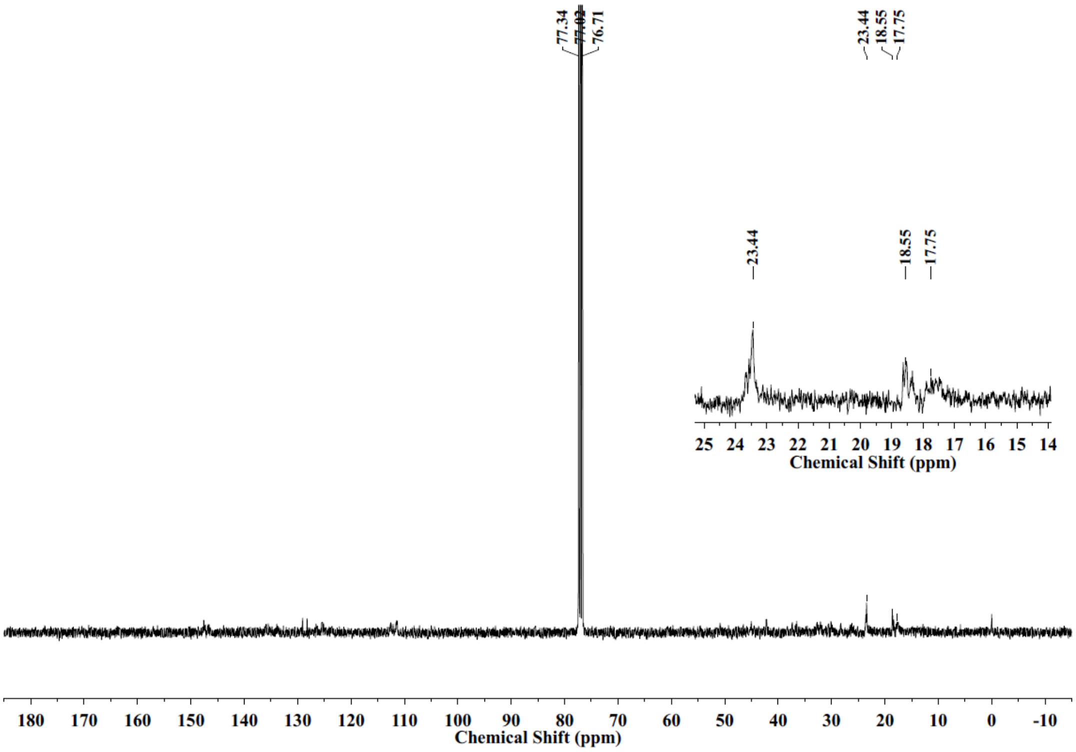

Table 1. entry 1 (^{13}C MMR).

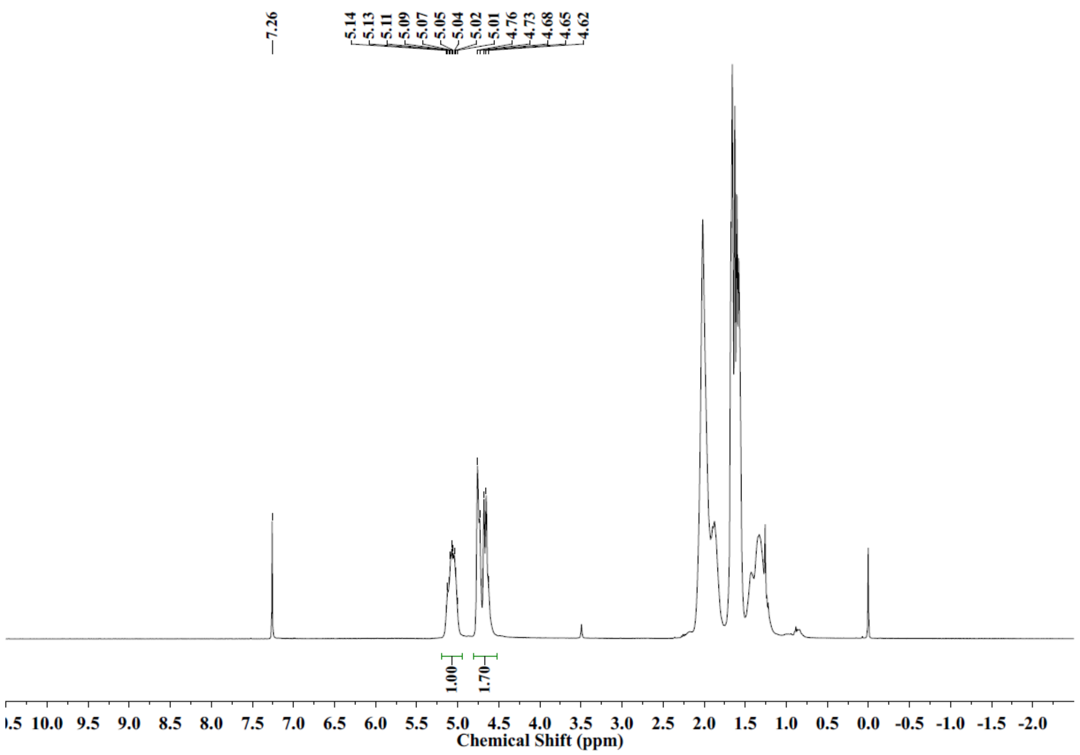

Table 1. entry 2 (^{1}H MMR).

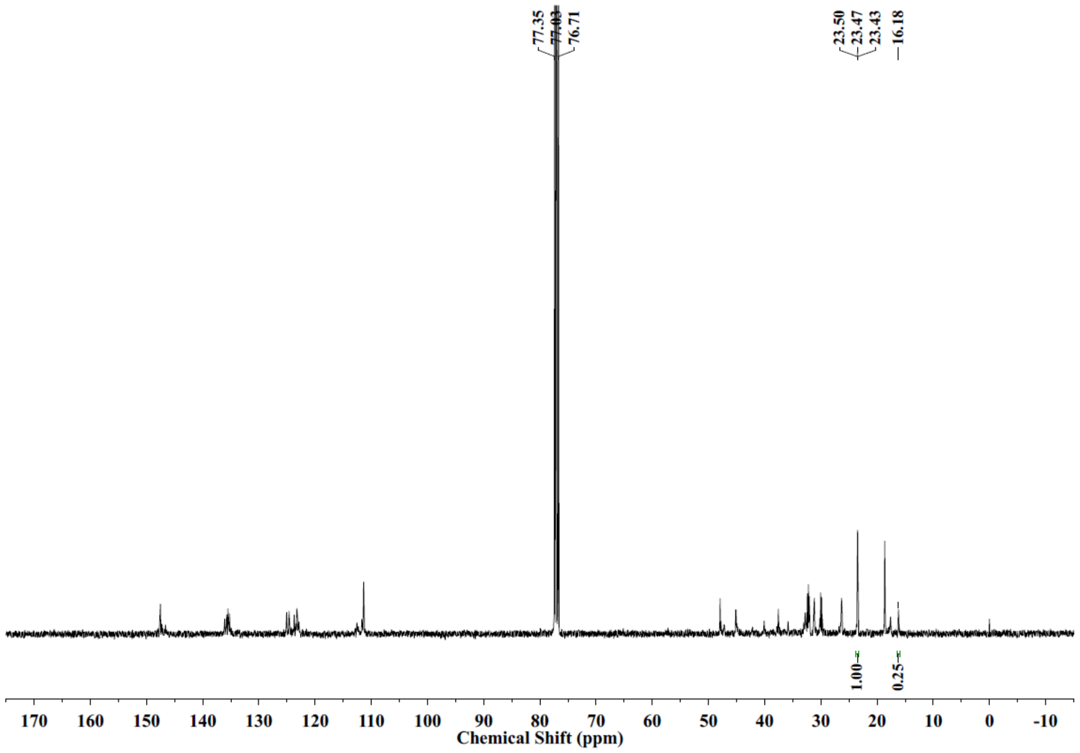

Table 1. entry 2 (^{13}C MMR).

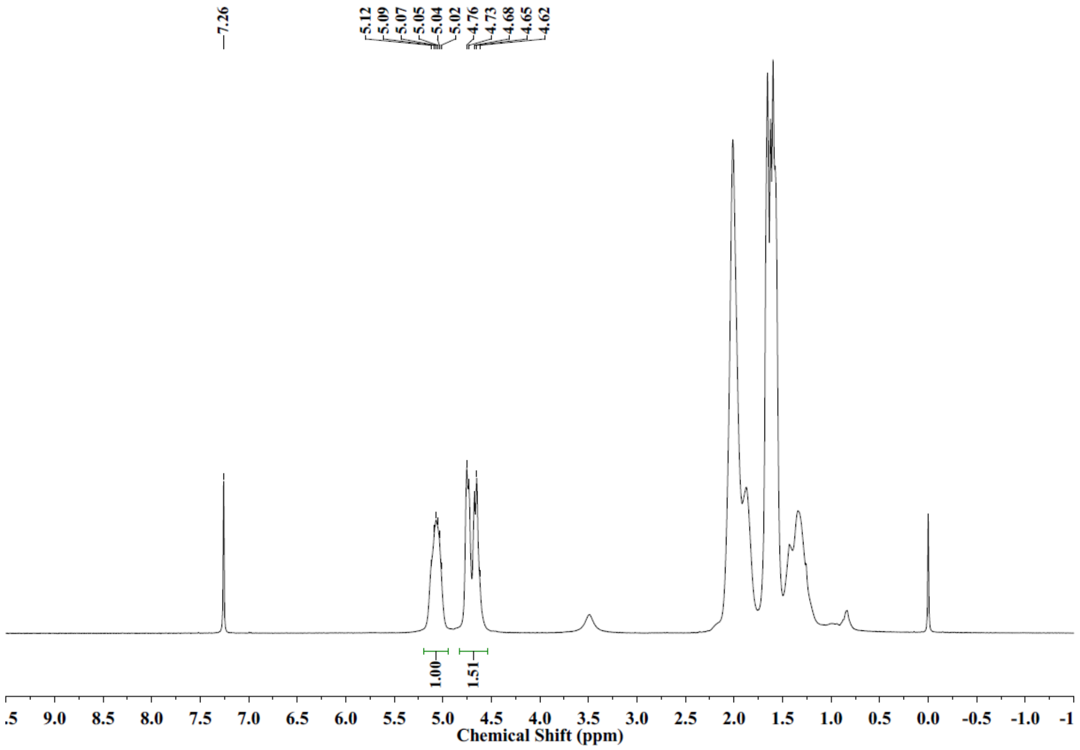

Table 1. entry 3 (^{1}H MMR).

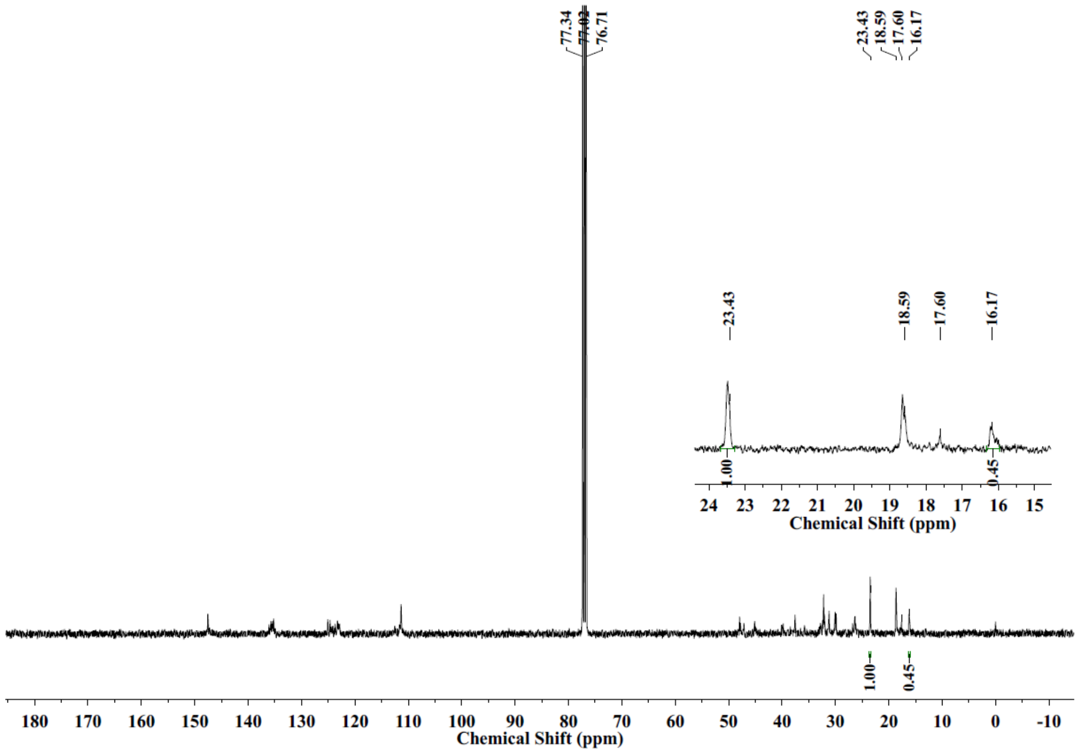

Table 1. entry 3 (^{13}C MMR).

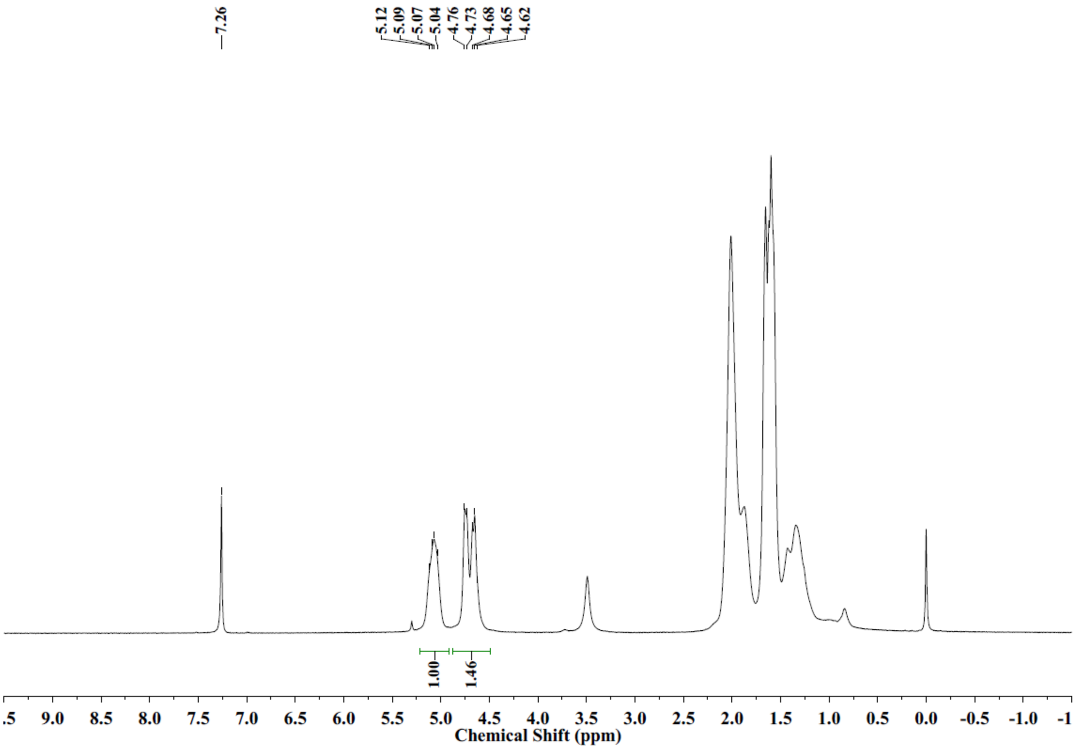

Table 1. entry 4 (^{1}H MMR).

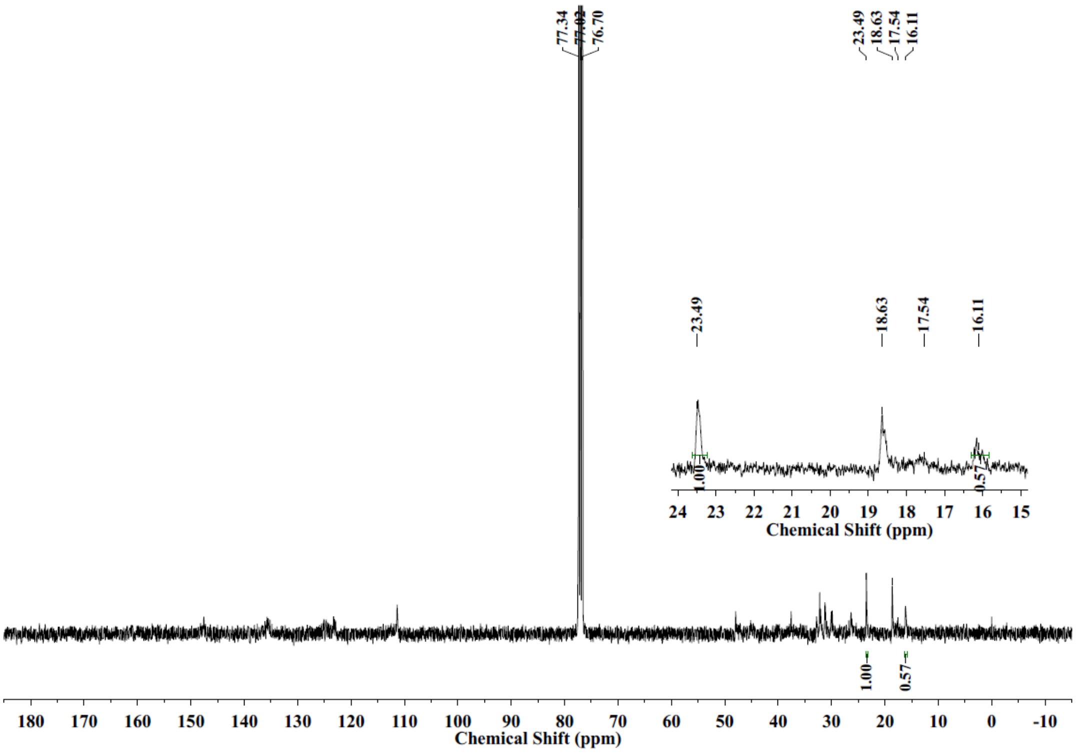

Table 1. entry 4 (^{13}C MMR).

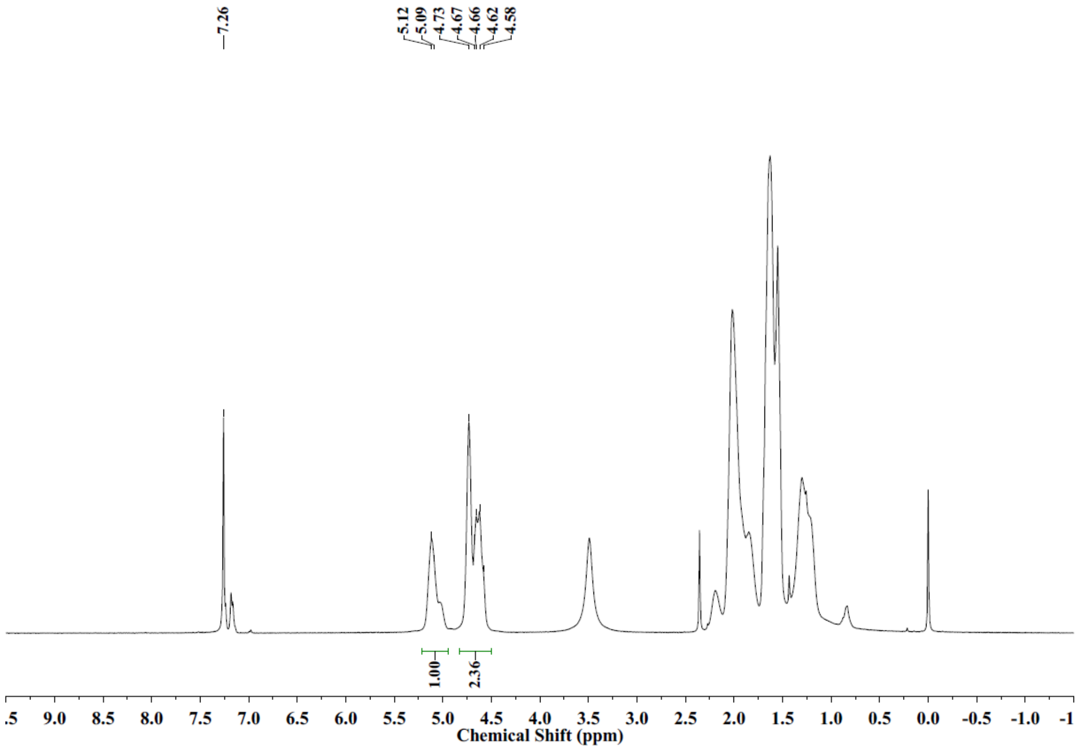

Table 1. entry 5 (^{1}H MMR).

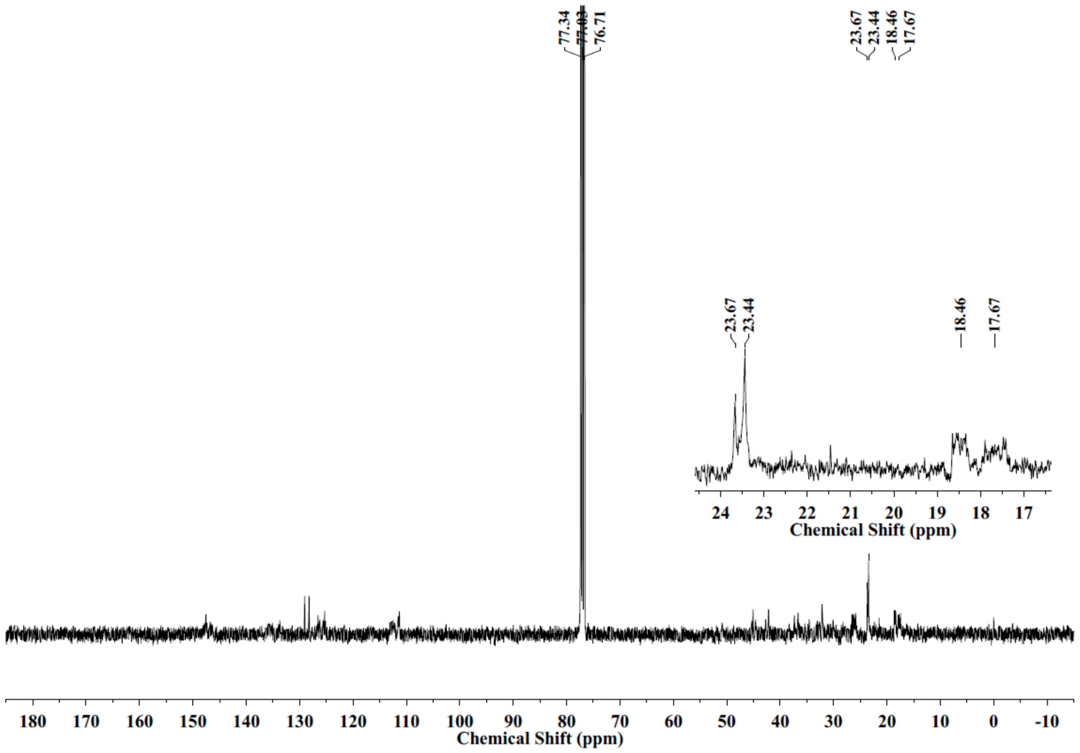

Table 1. entry 5 (^{13}C MMR).

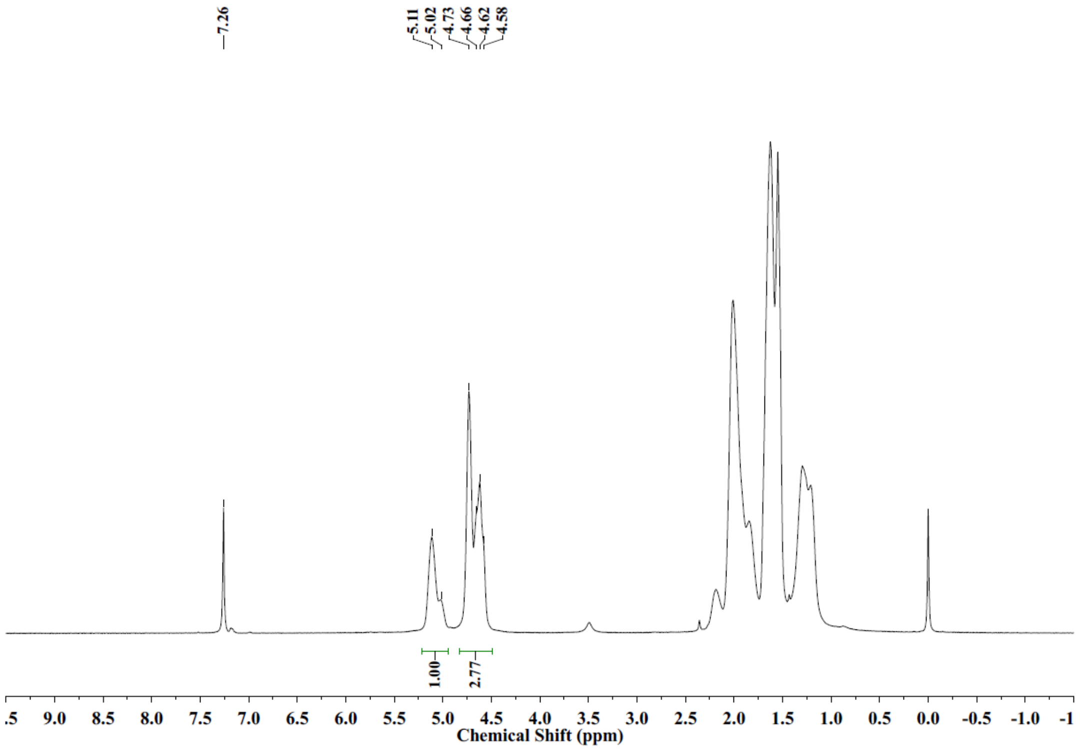

Table 1. entry 6 (^{1}H MMR).

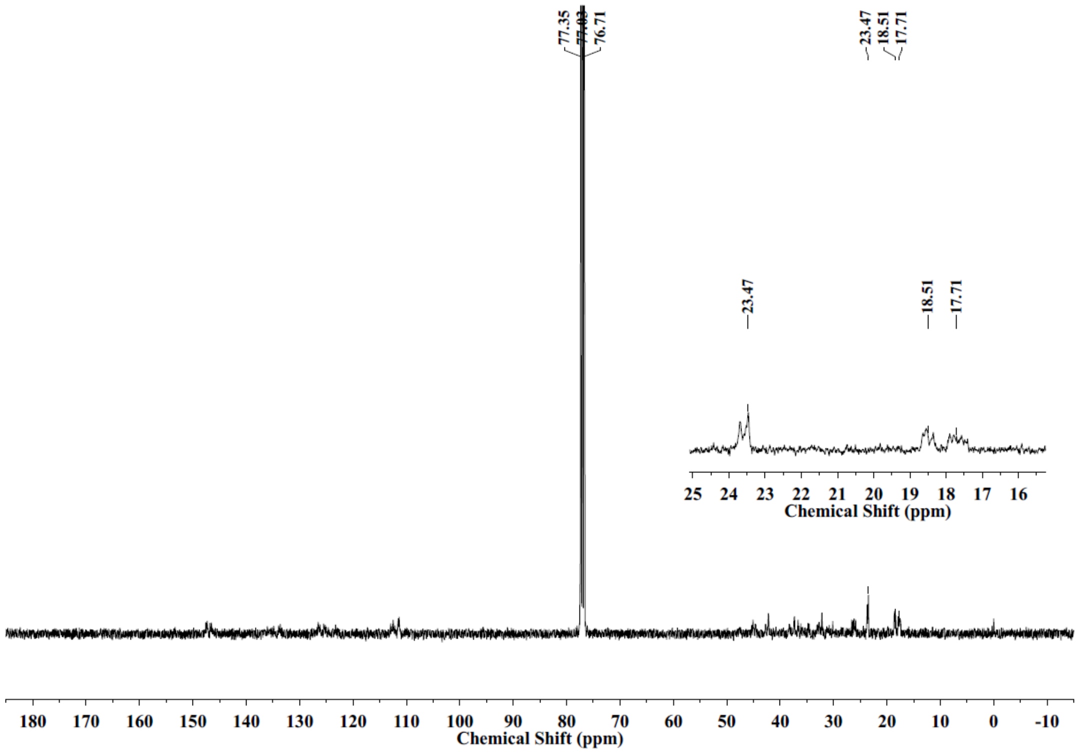

Table 1. entry 6 (^{13}C MMR).

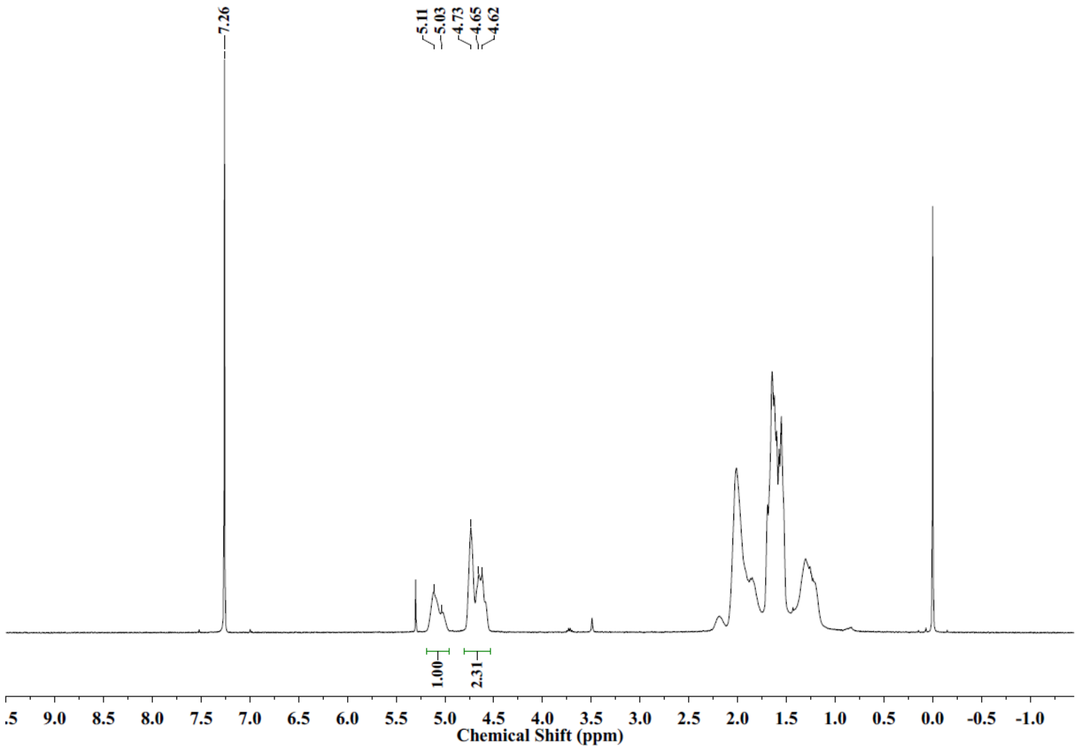

Table 2. entry 1 (^{1}H MMR).

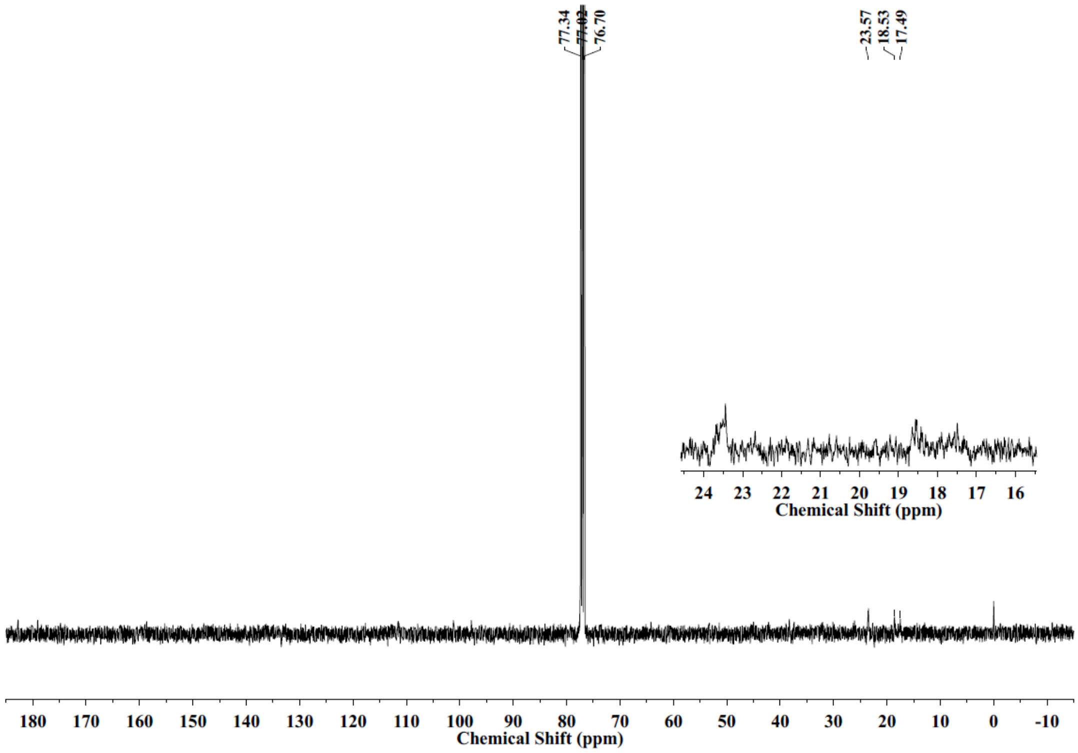

Table 2. entry 1 (^{13}C MMR).

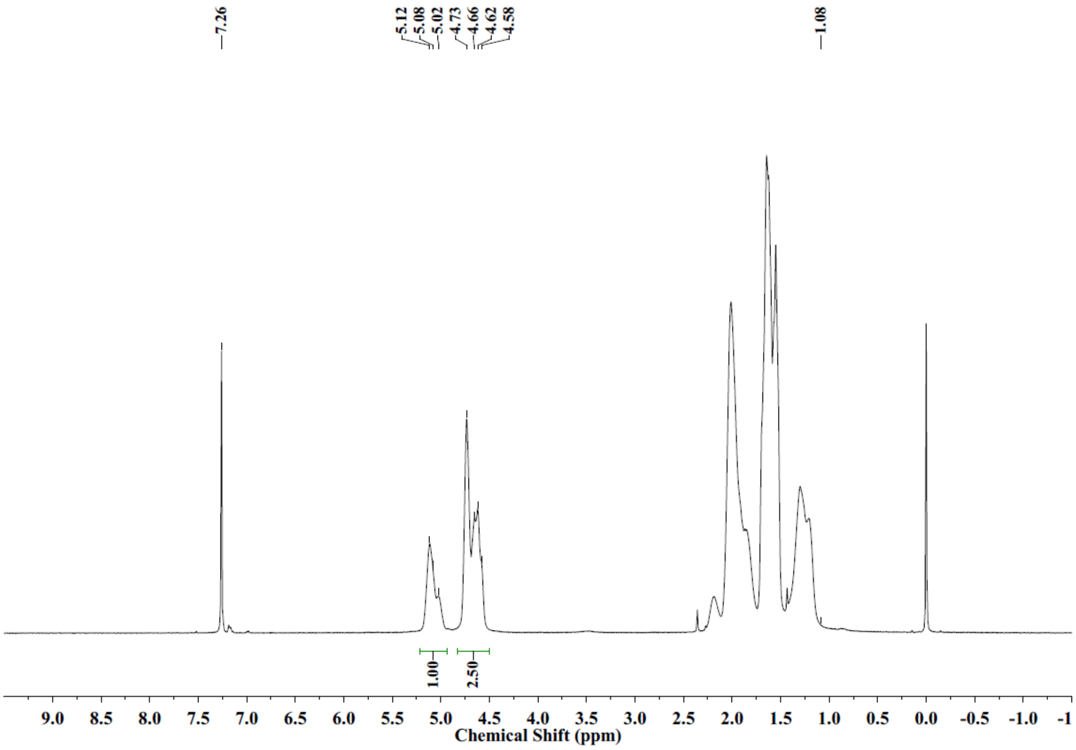

Table 2. entry 2 (^{1}H MMR).

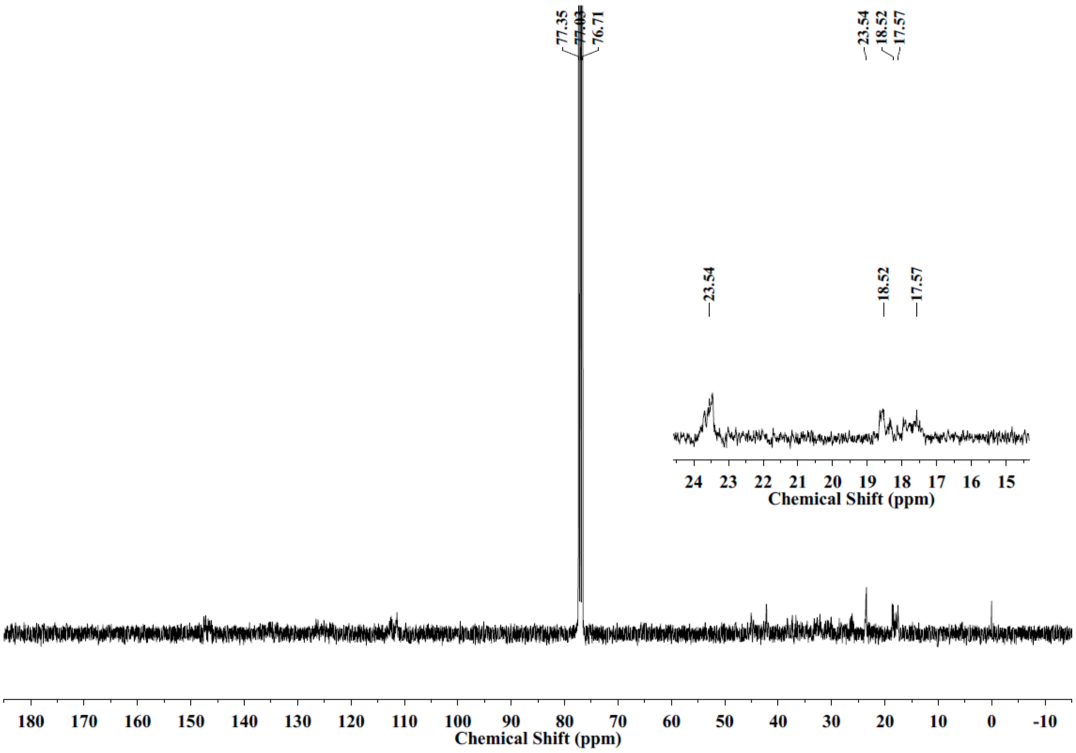

Table 2. entry 2 (^{13}C MMR).

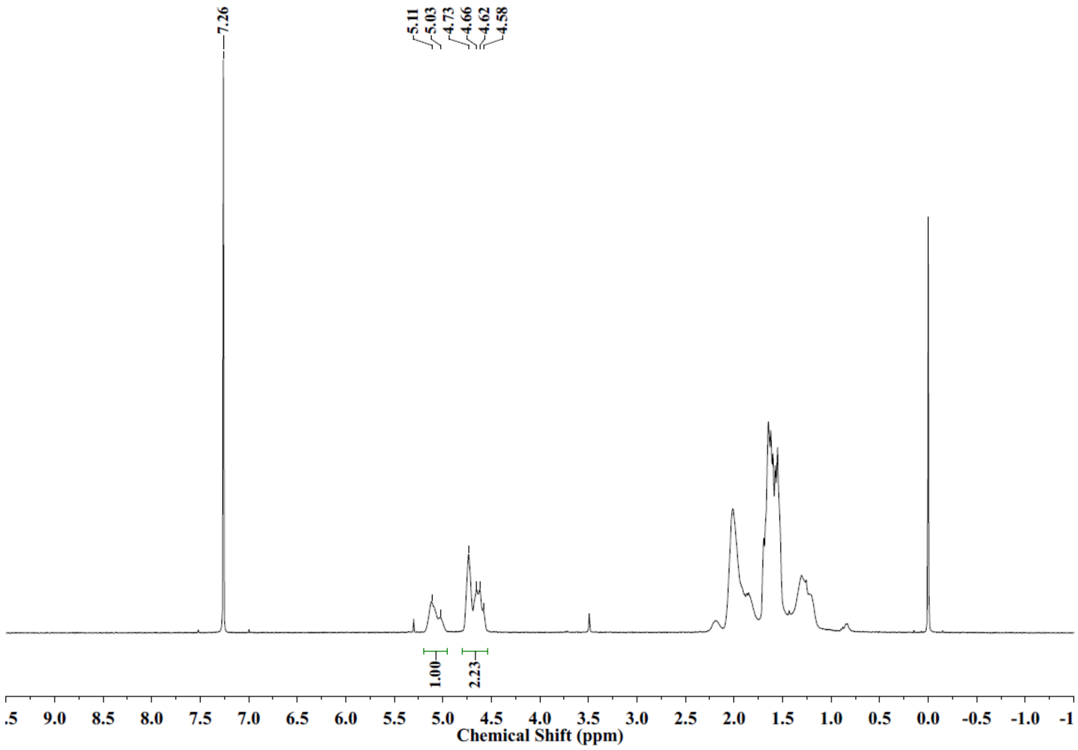

Table 2. entry 3 (¹H MMR).

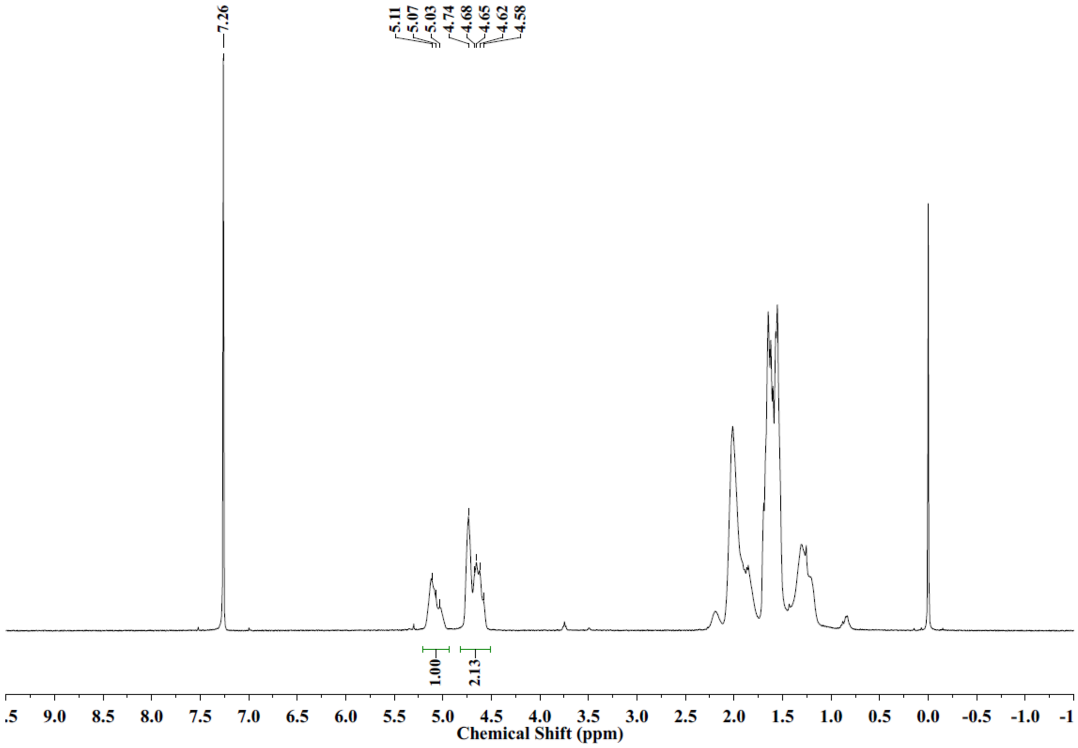

Table 2. entry 4 (¹H MMR).

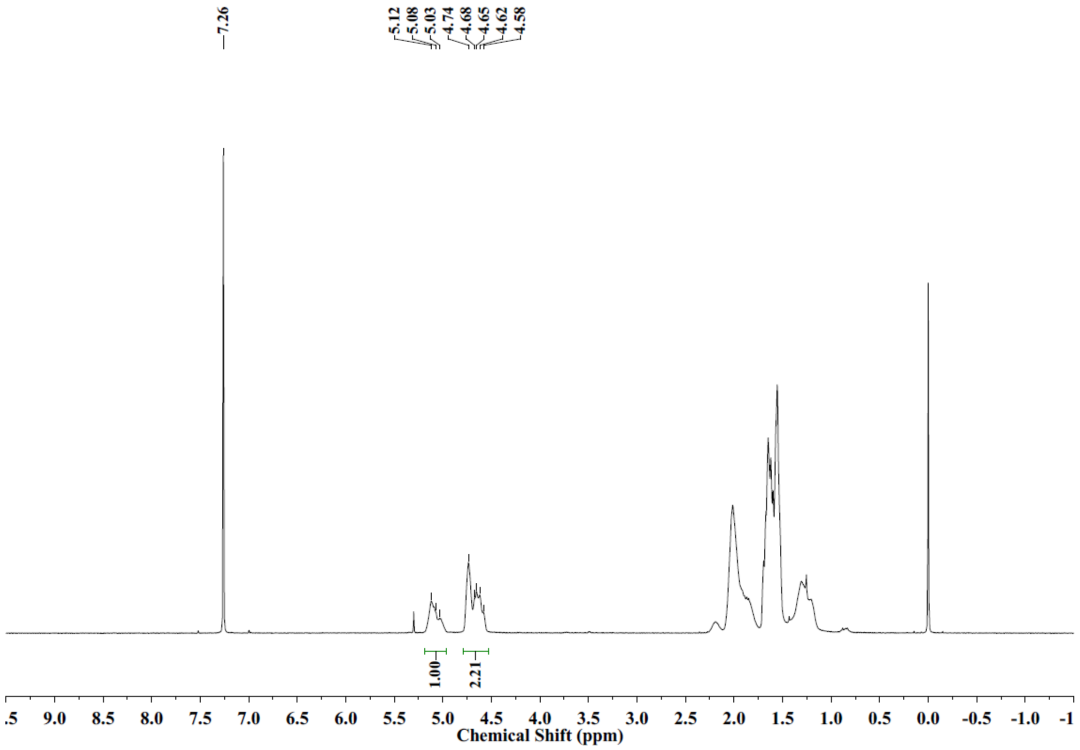

Table 2. entry 5 (^{1}H MMR).

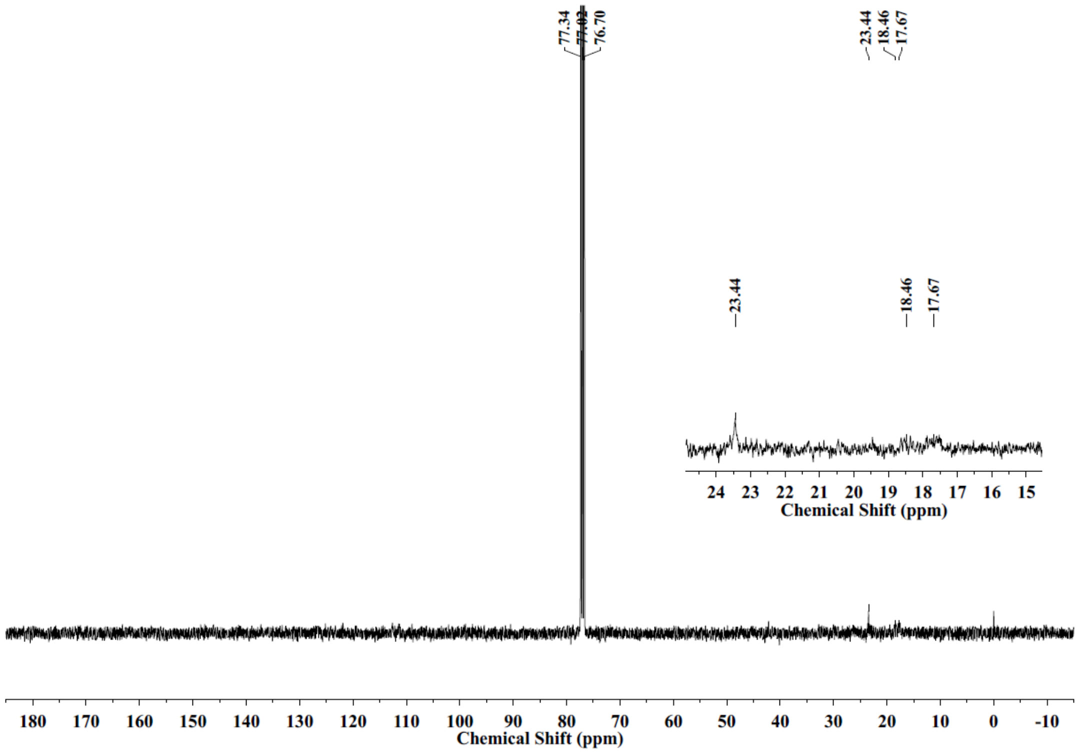

Table 2. entry 5 (^{13}C MMR).

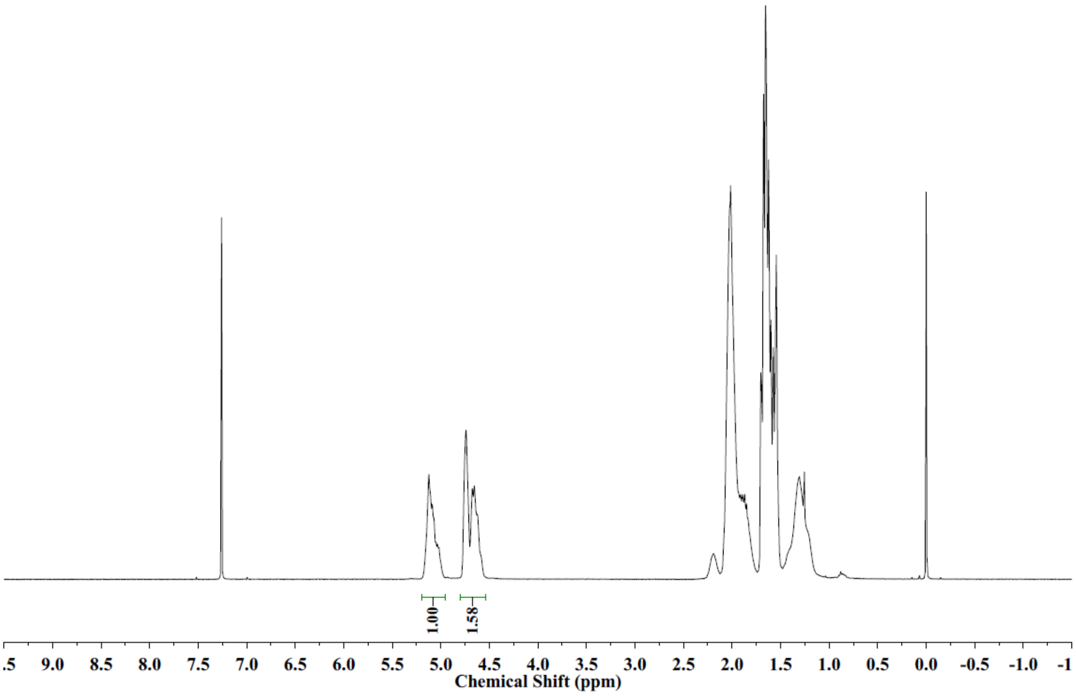

Table 2. entry 6 (^{1}H MMR).

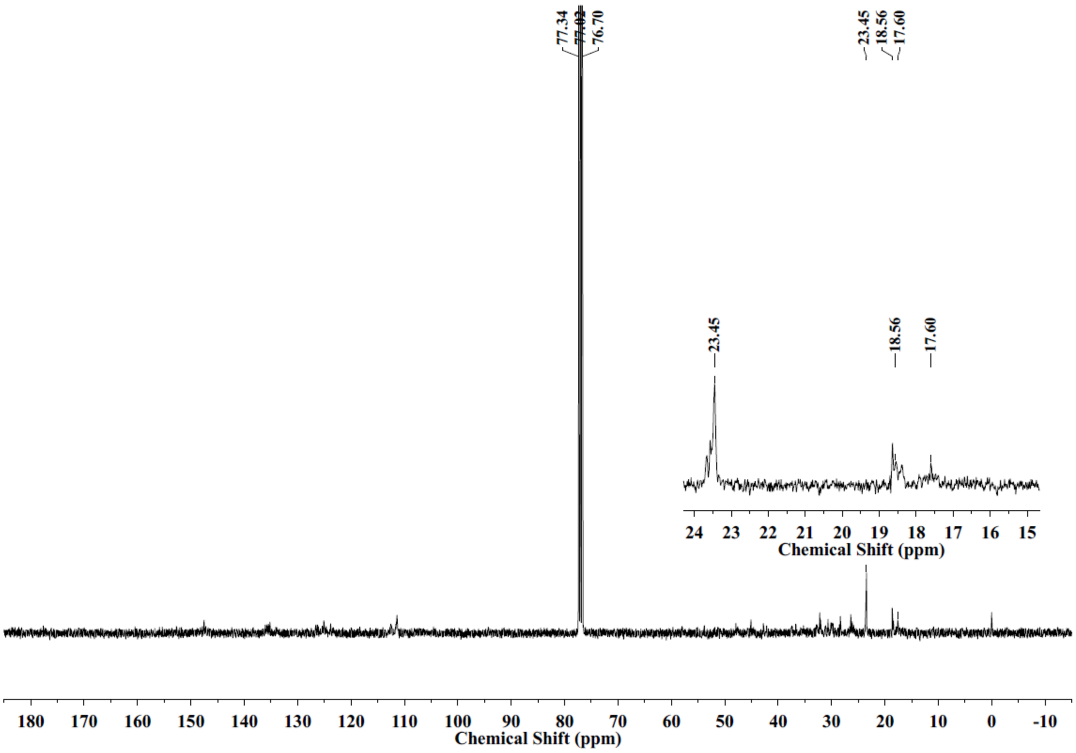

Table 2. entry 6 (^{13}C MMR).

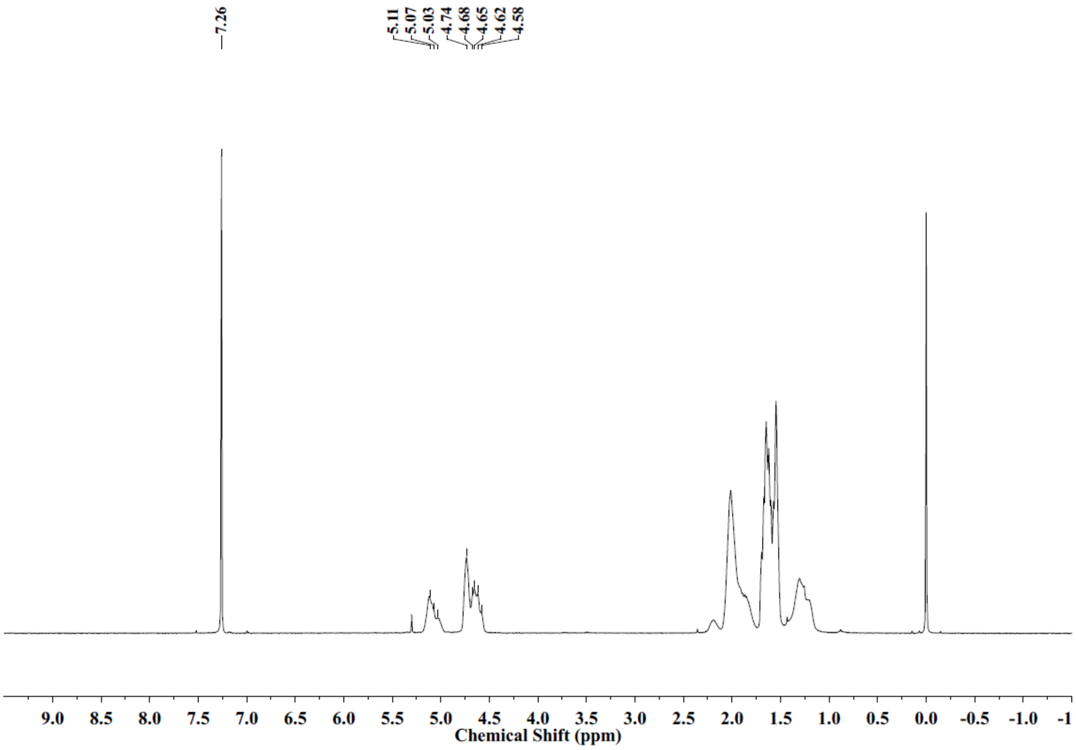

Table 2. entry 7 (^{1}H MMR).

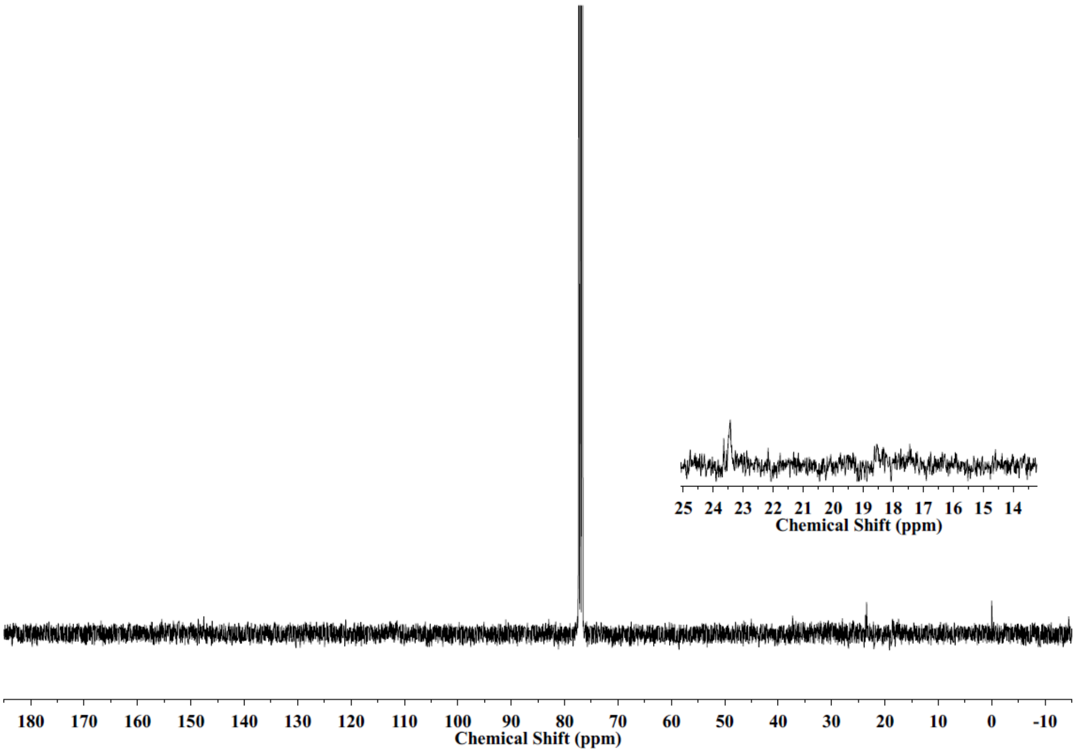

Table 2. entry 7 (^{13}C MMR).

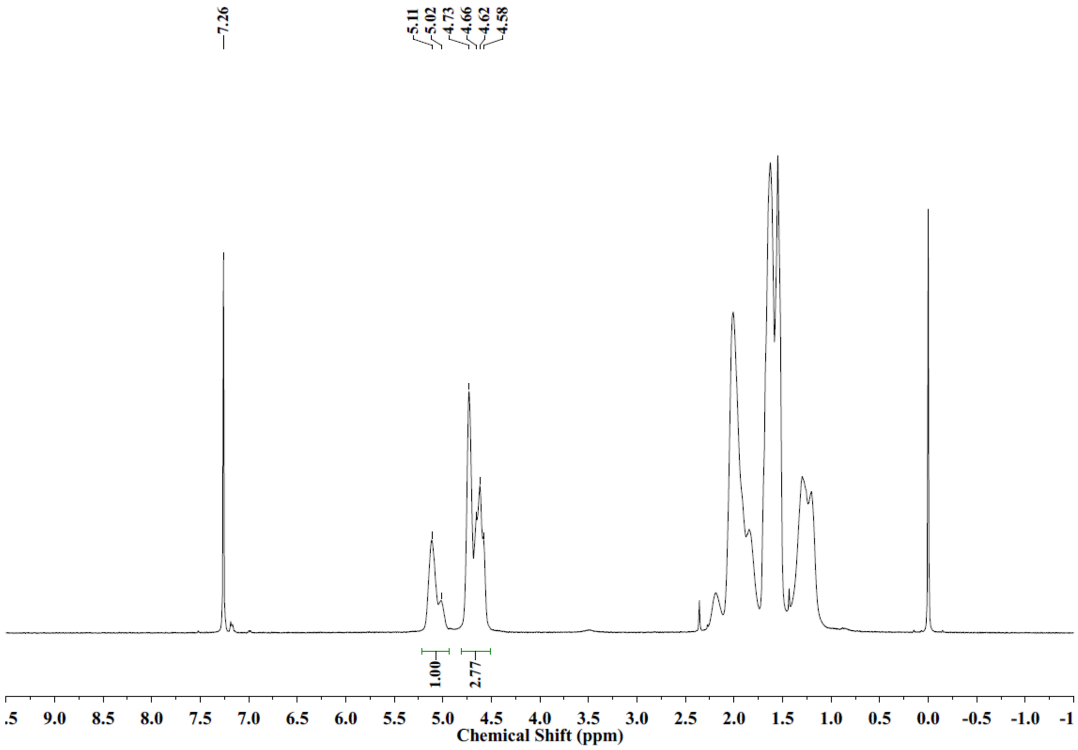

Table 2. entry 8 (^{1}H MMR).

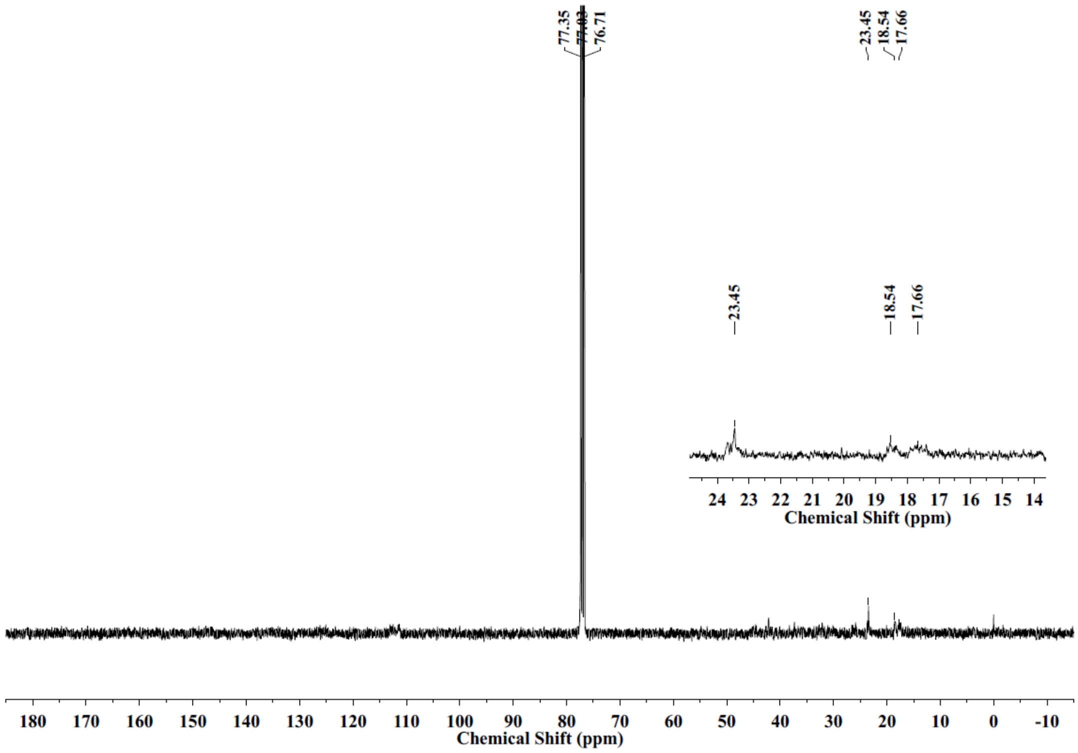

Table 2. entry 8 (^{13}C MMR).

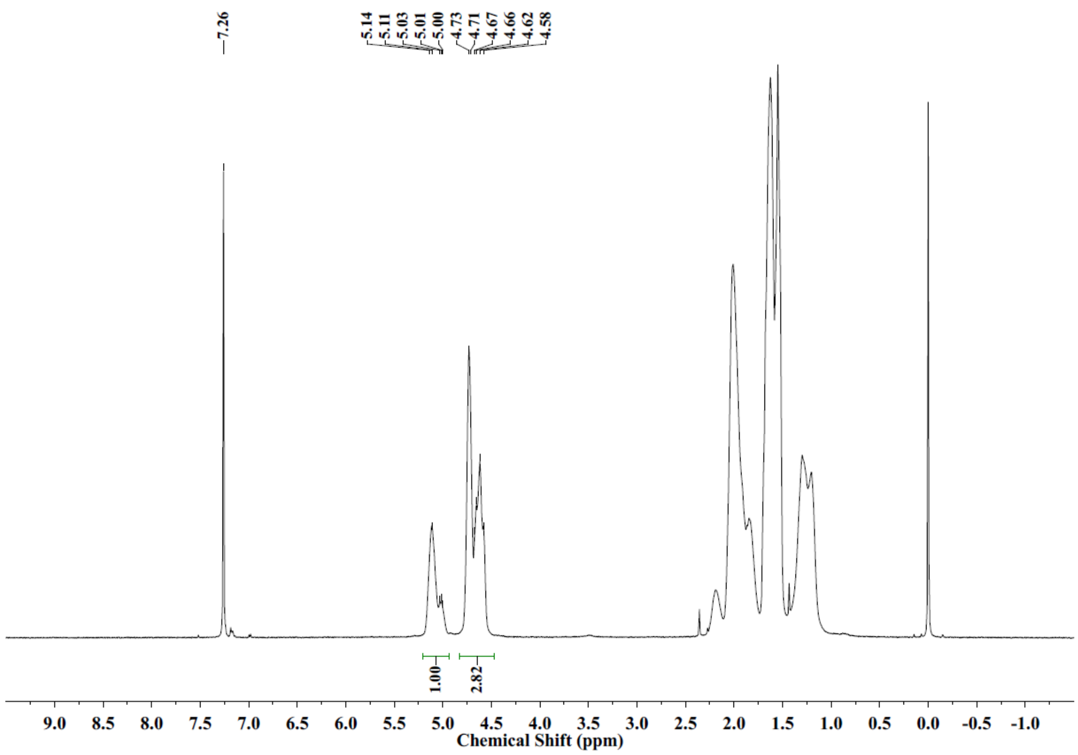

Table 2. entry 9 (^{1}H MMR).

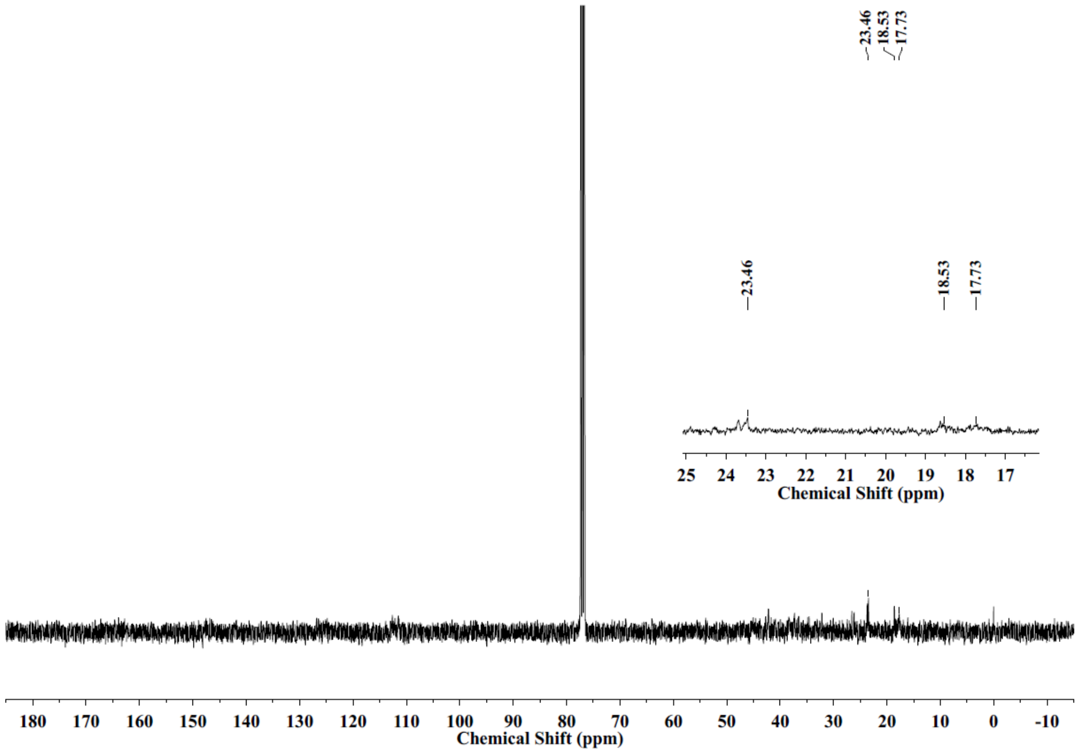

Table 2. entry 9 (^{13}C MMR).

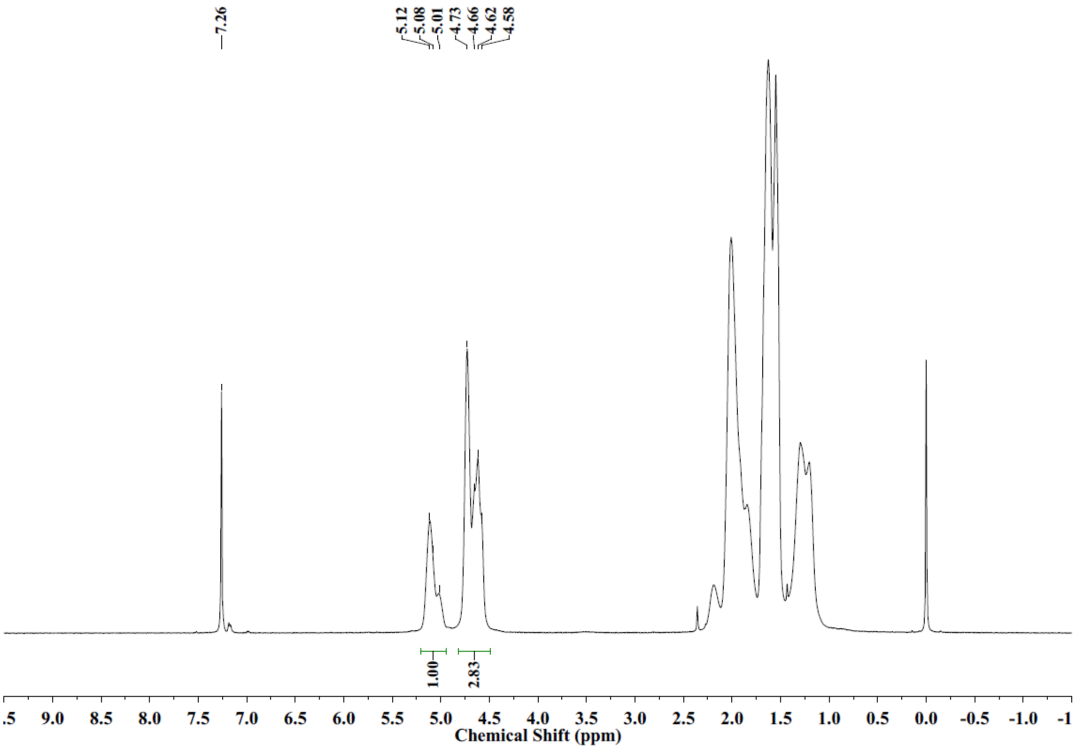

Table 2. entry 10 (^{1}H MMR).

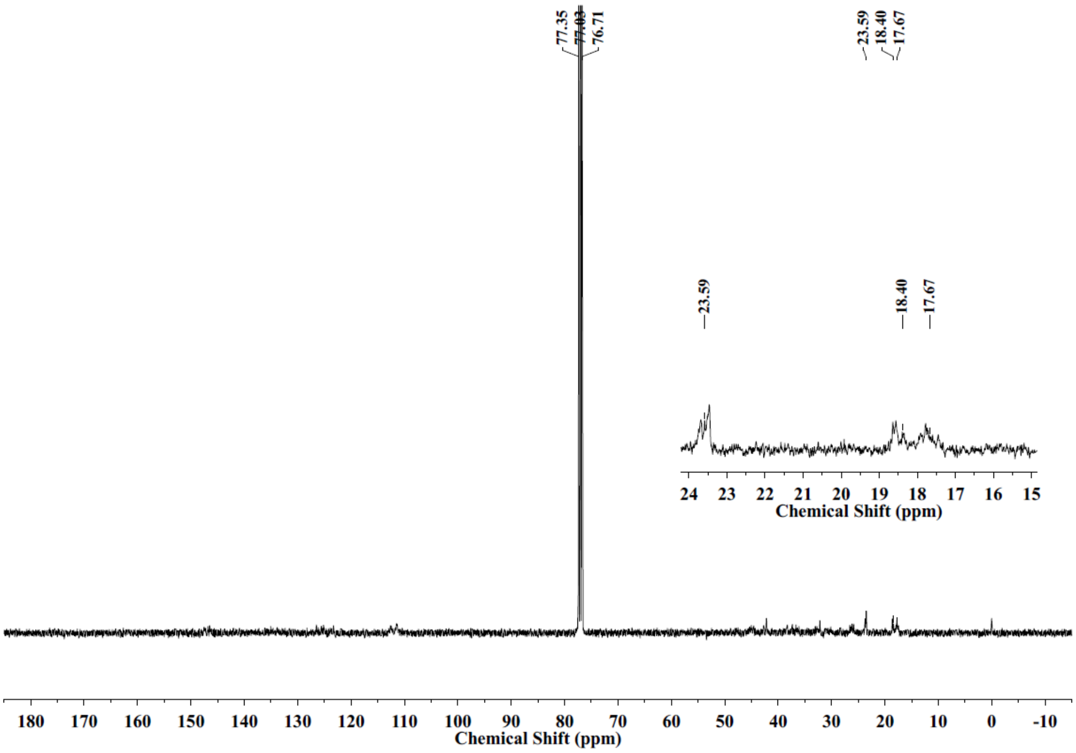

Table 2. entry 10 (^{13}C MMR).

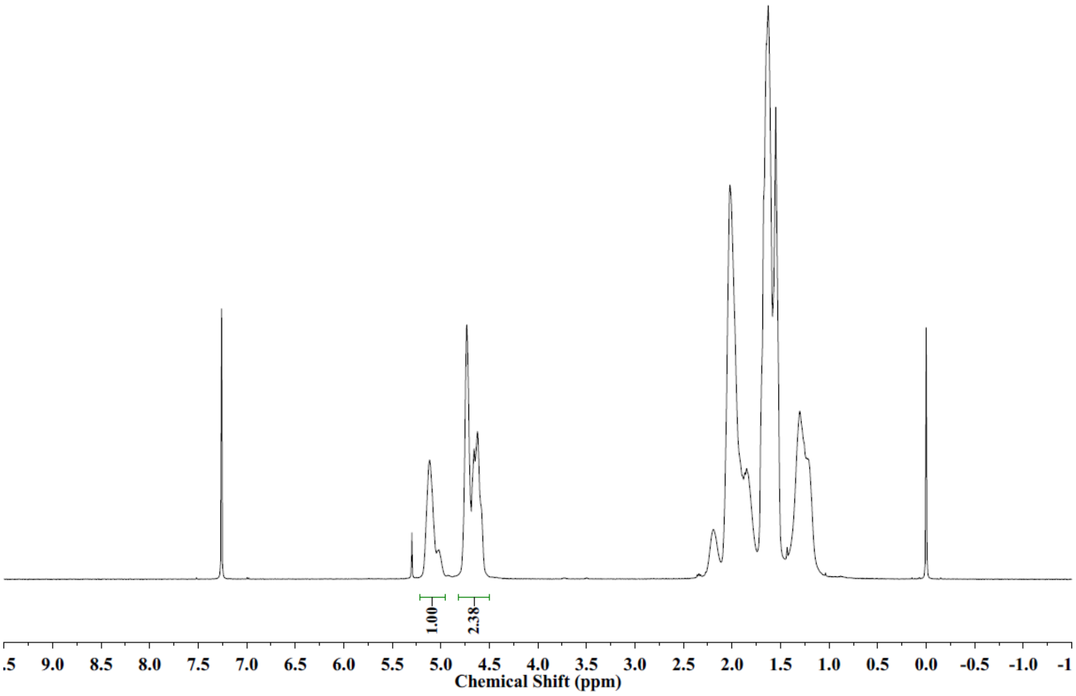

Table 2. entry 11 (^{1}H MMR).

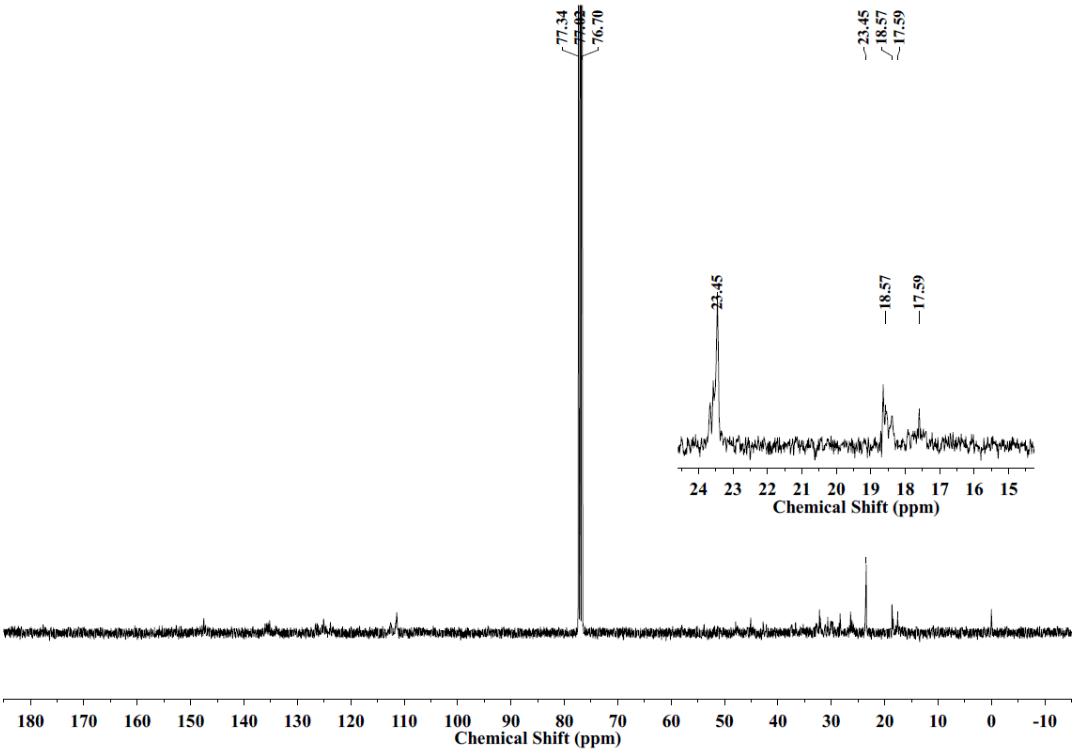

Table 2. entry 11 (^{13}C MMR).

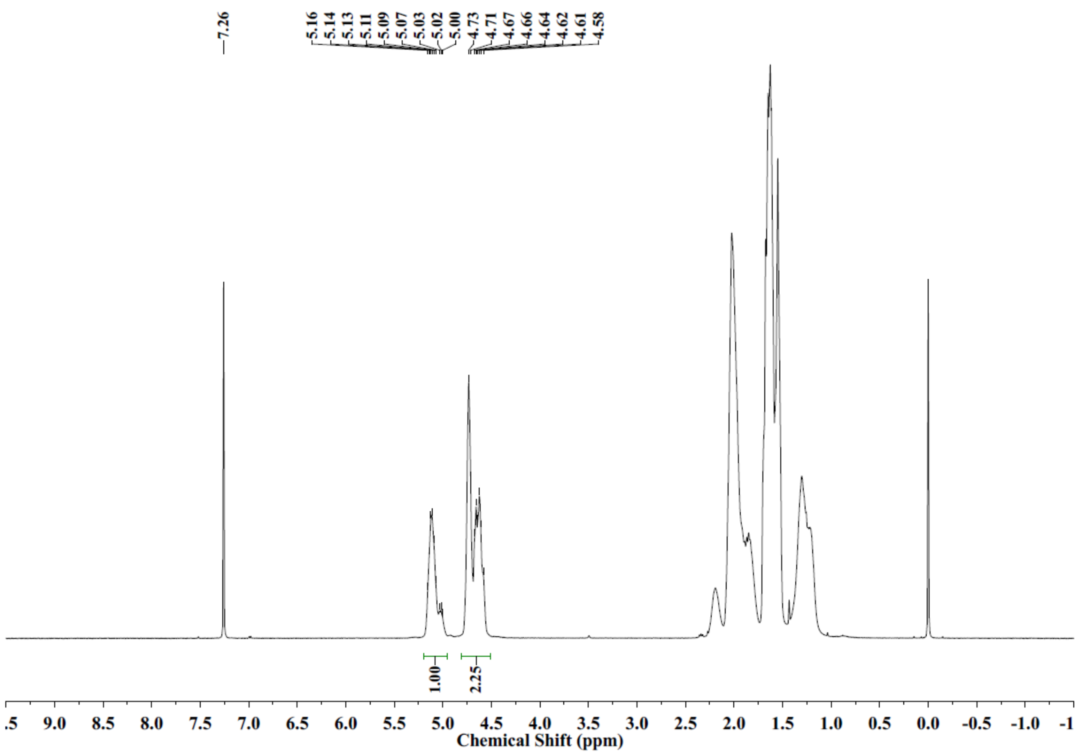

Table 2. entry 12 (^{1}H MMR).

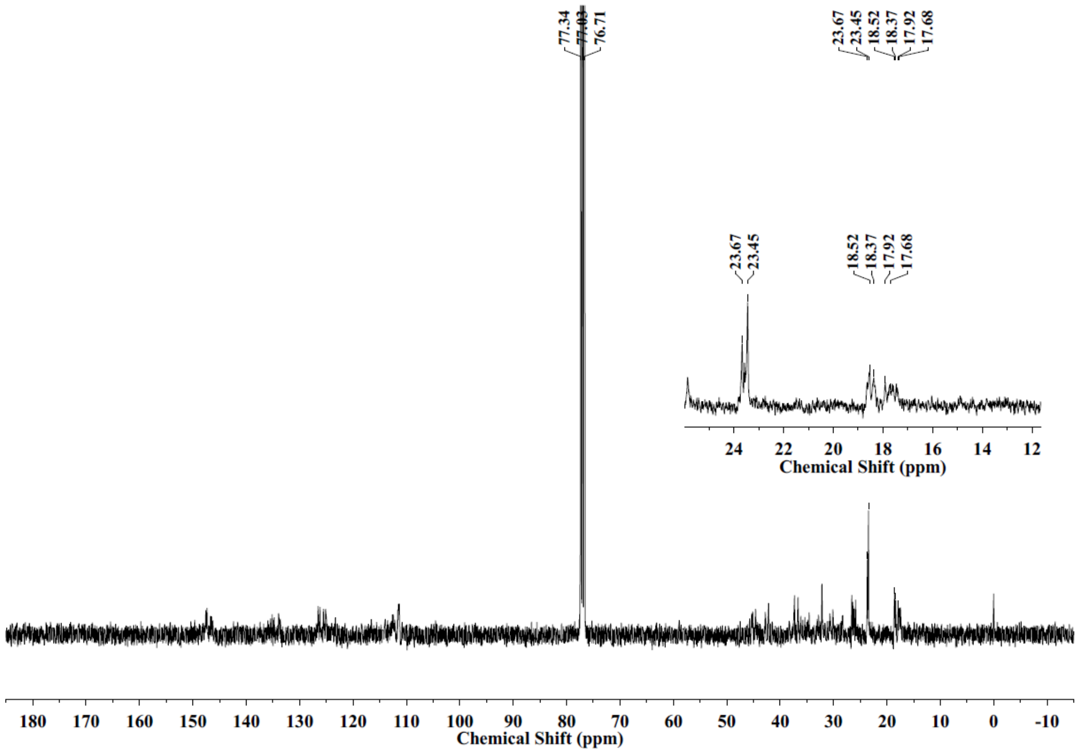

Table 2. entry 12 (^{13}C MMR).

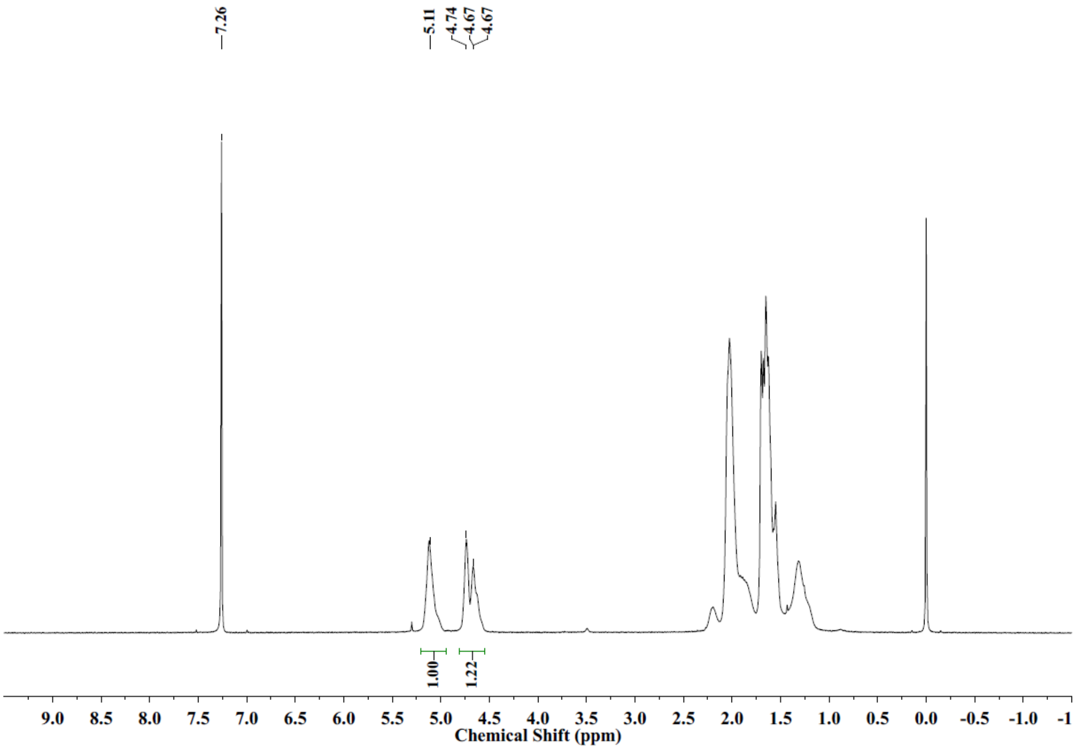

Table 2. entry 13 (^1H MMR).

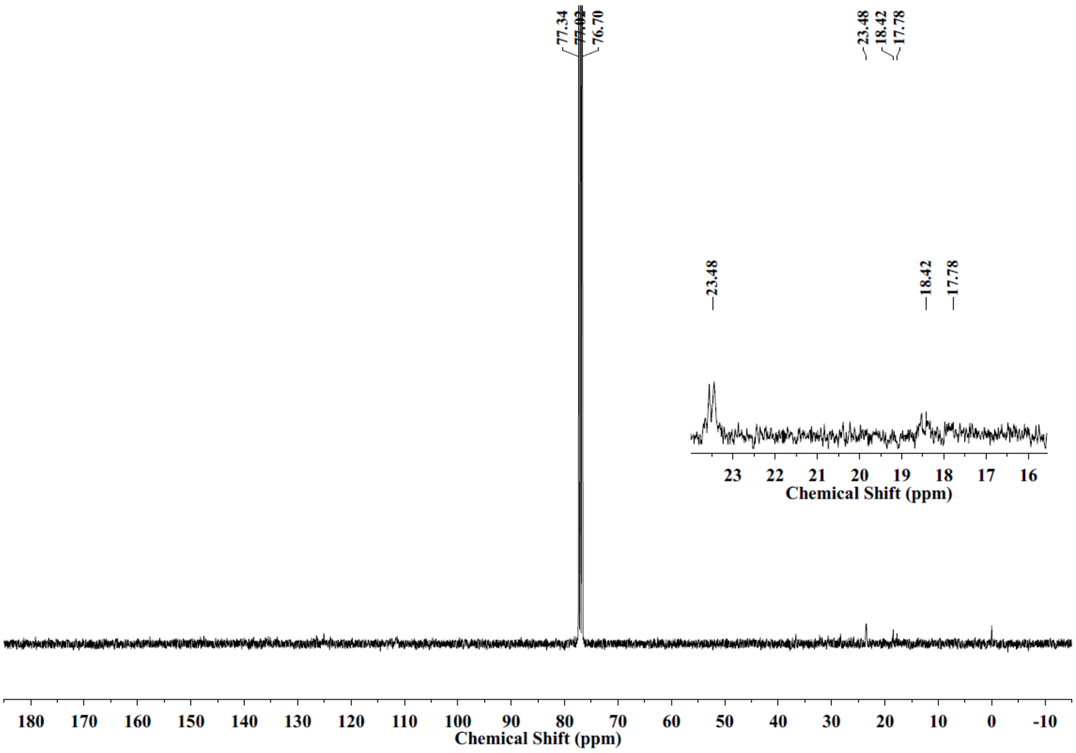

Table 2. entry 13 (^{13}C MMR).

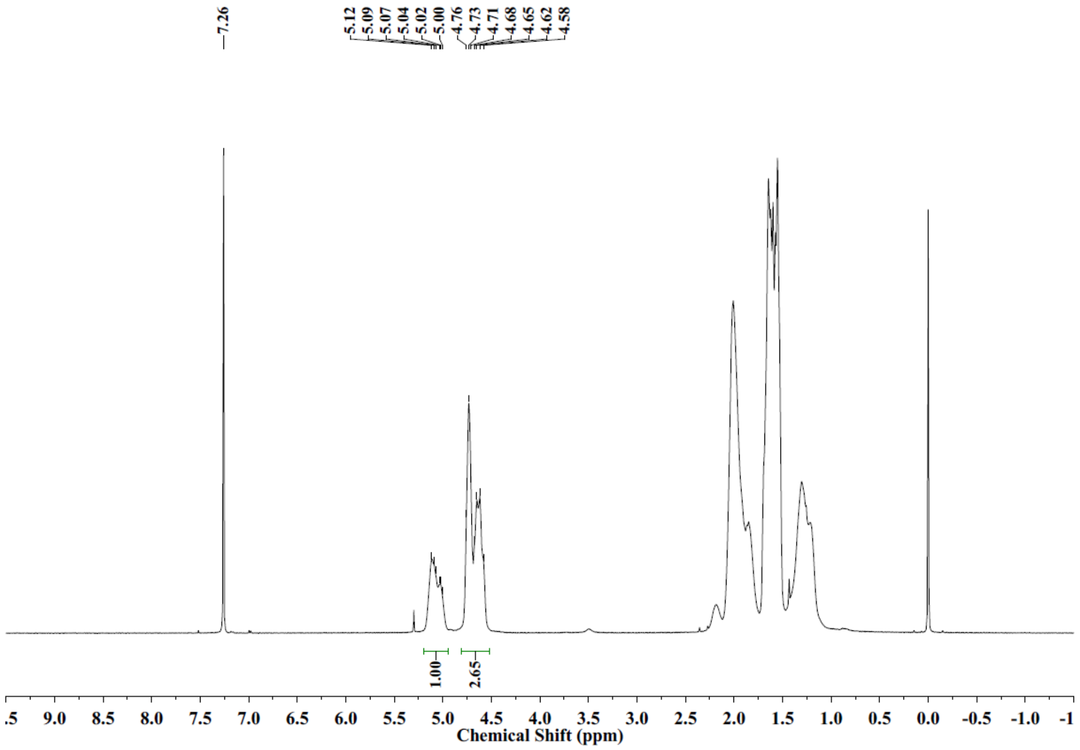

Table 2. entry 14 (^{1}H MMR).

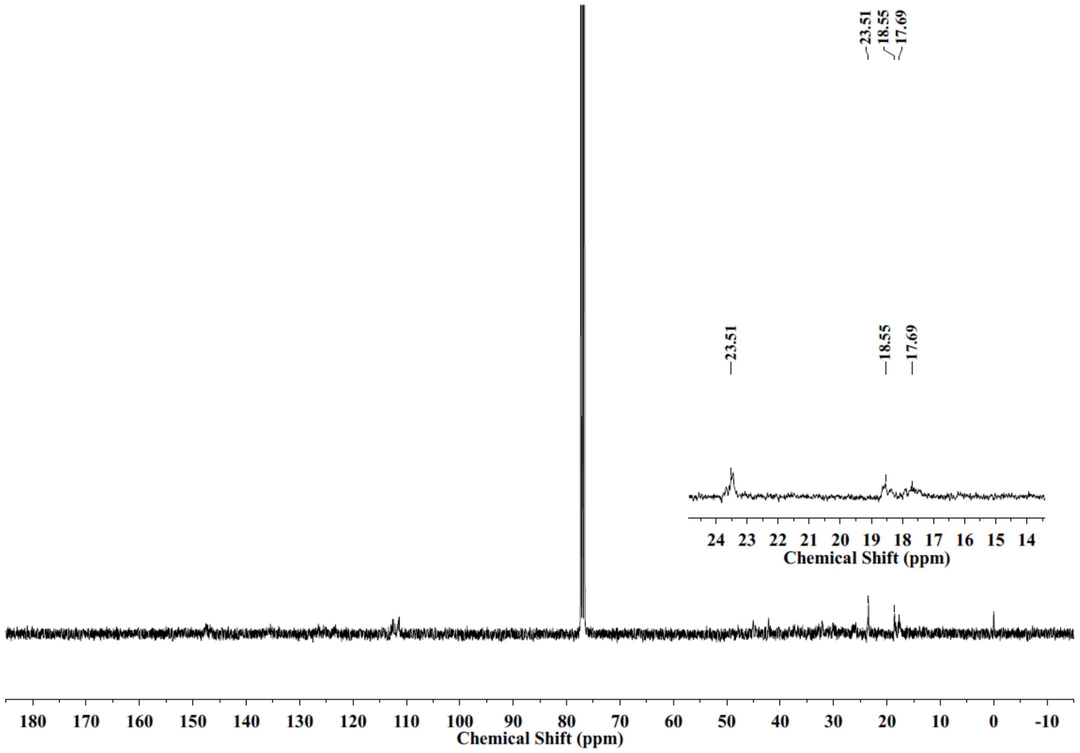

Table 2. entry 14 (^{13}C MMR).

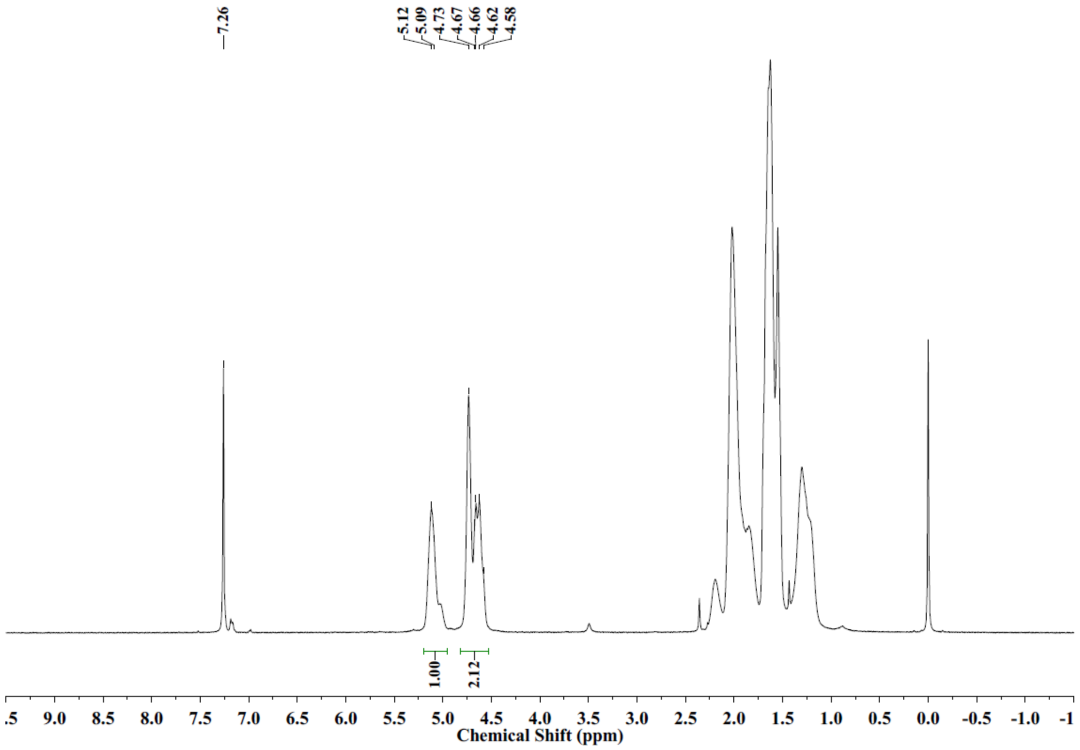

Table 2. entry 15 (^{1}H MMR).

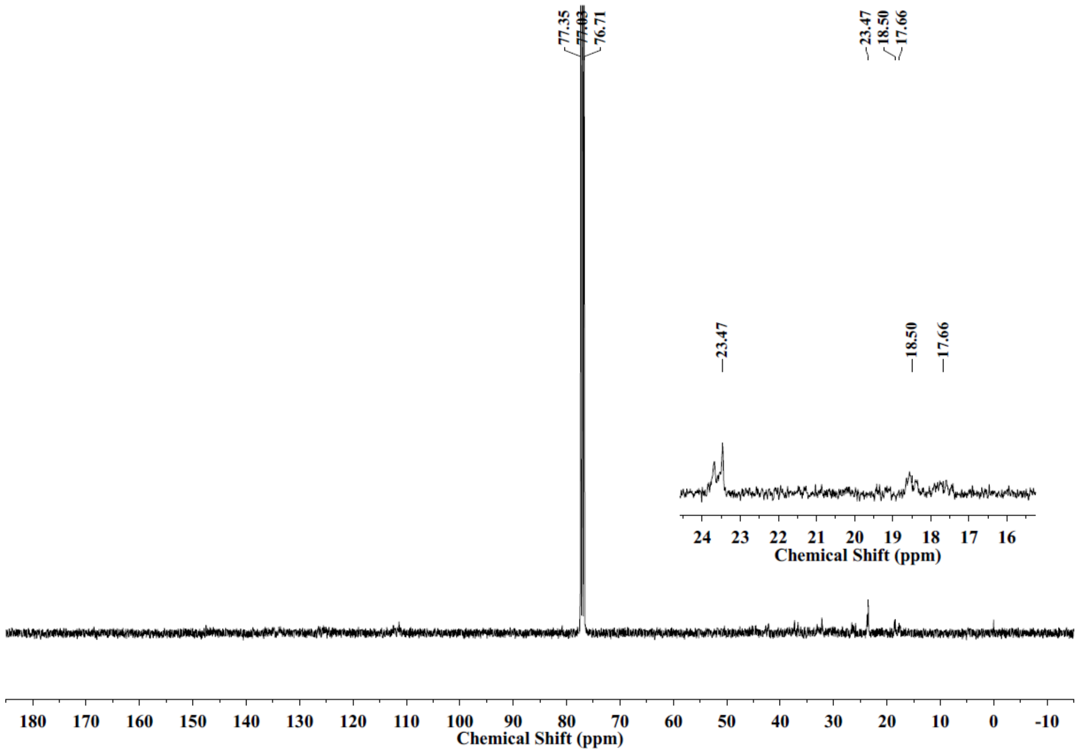

Table 2. entry 15 (^{13}C MMR).

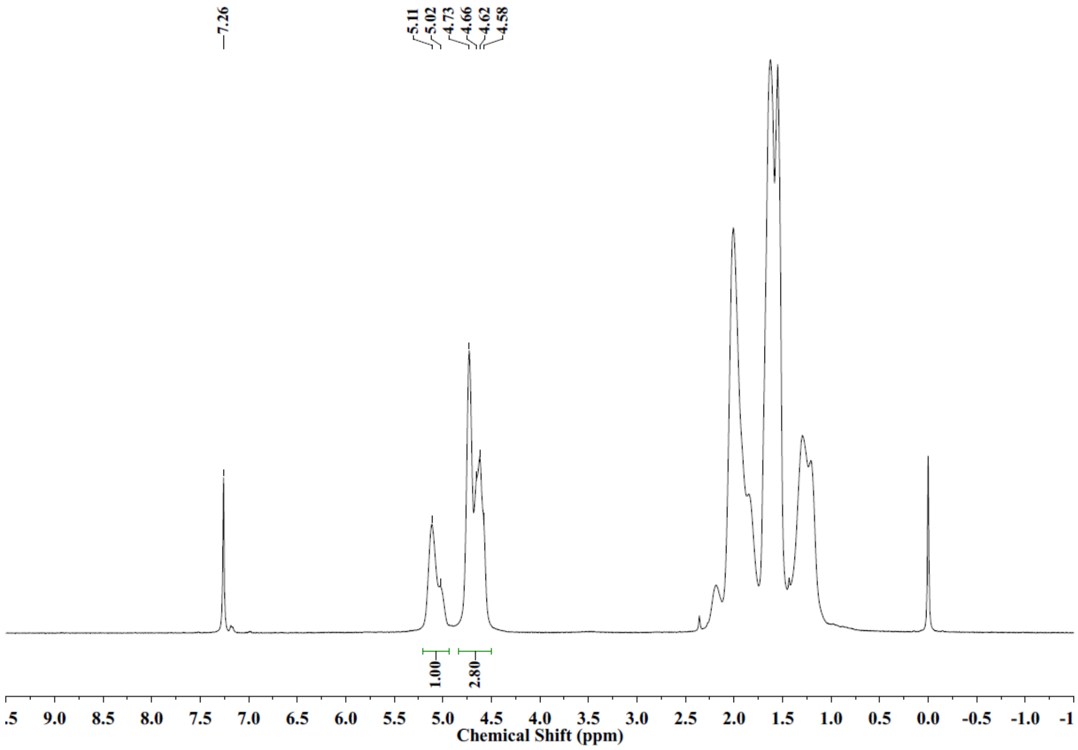

Table 2. entry 16 (^{1}H MMR).

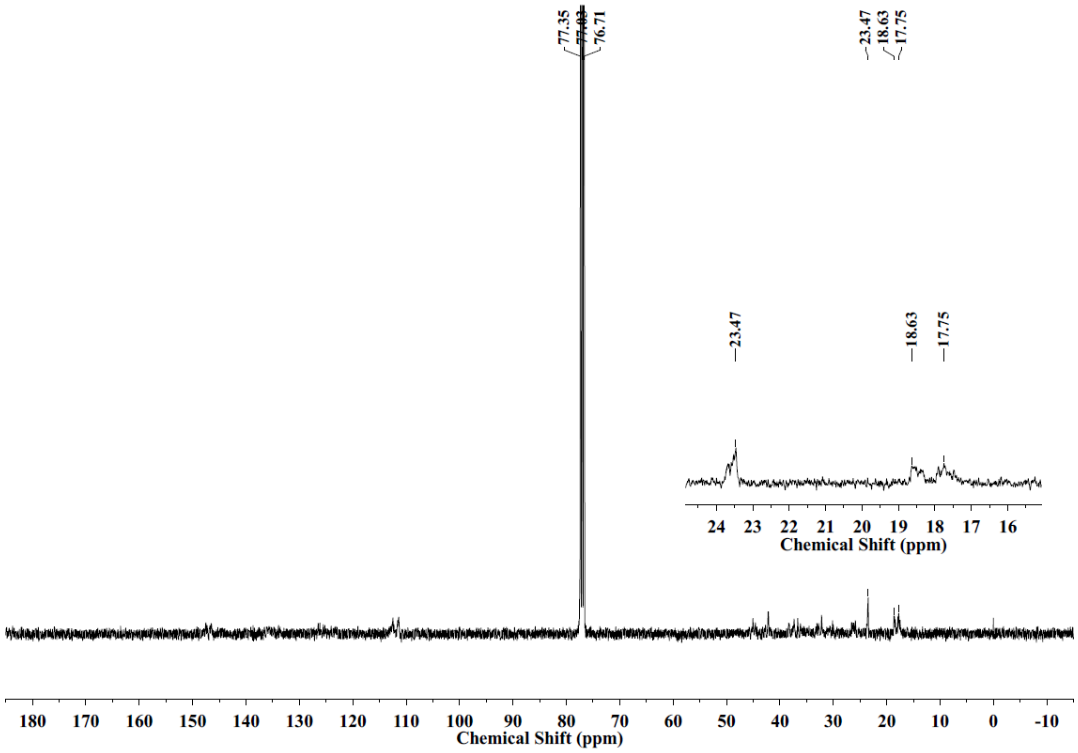

Table 2. entry 16 (^{13}C MMR).

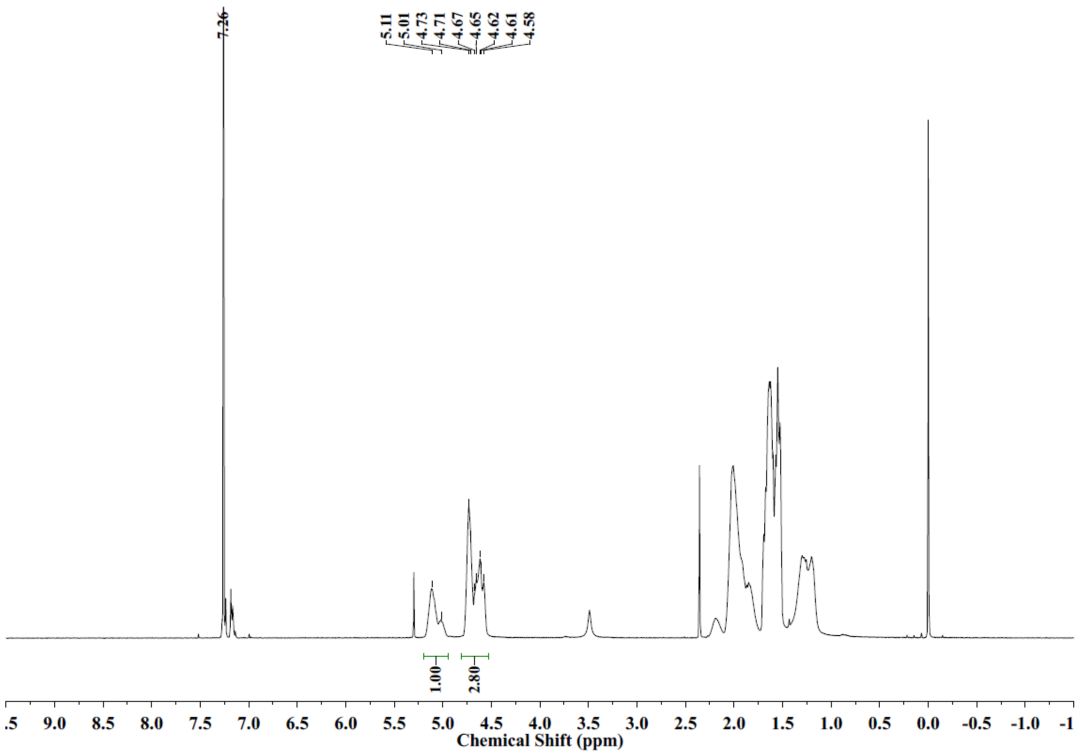

Table 2. entry 17 (^{1}H MMR).

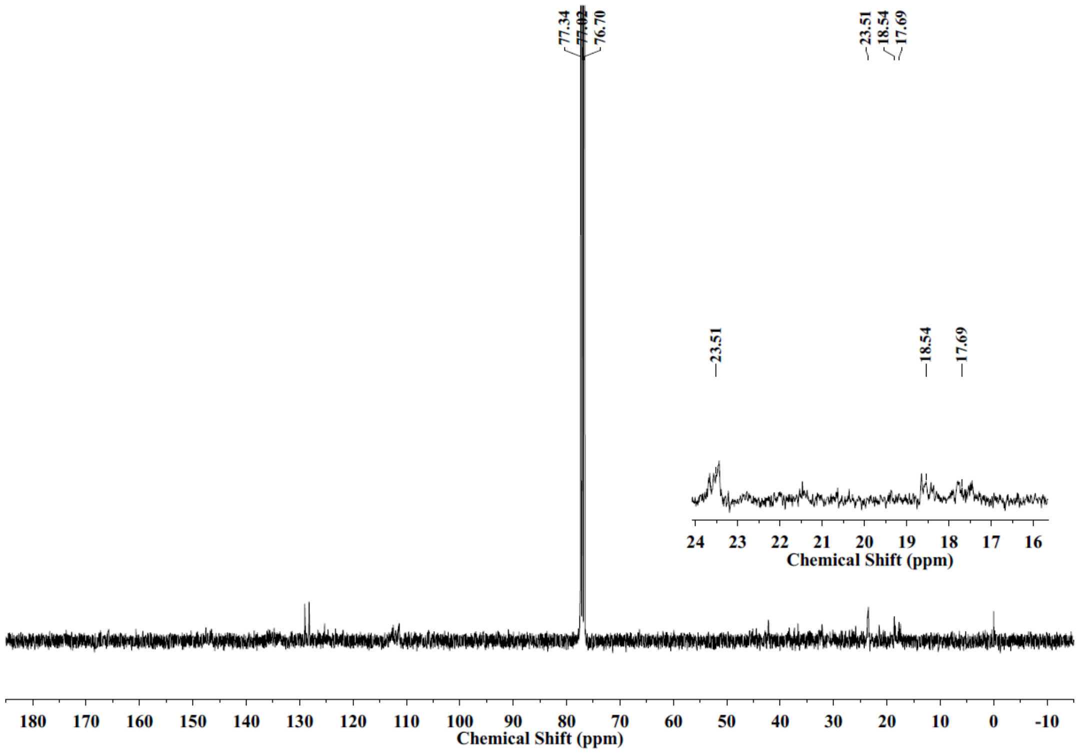

Table 2. entry 17 (^{13}C MMR).

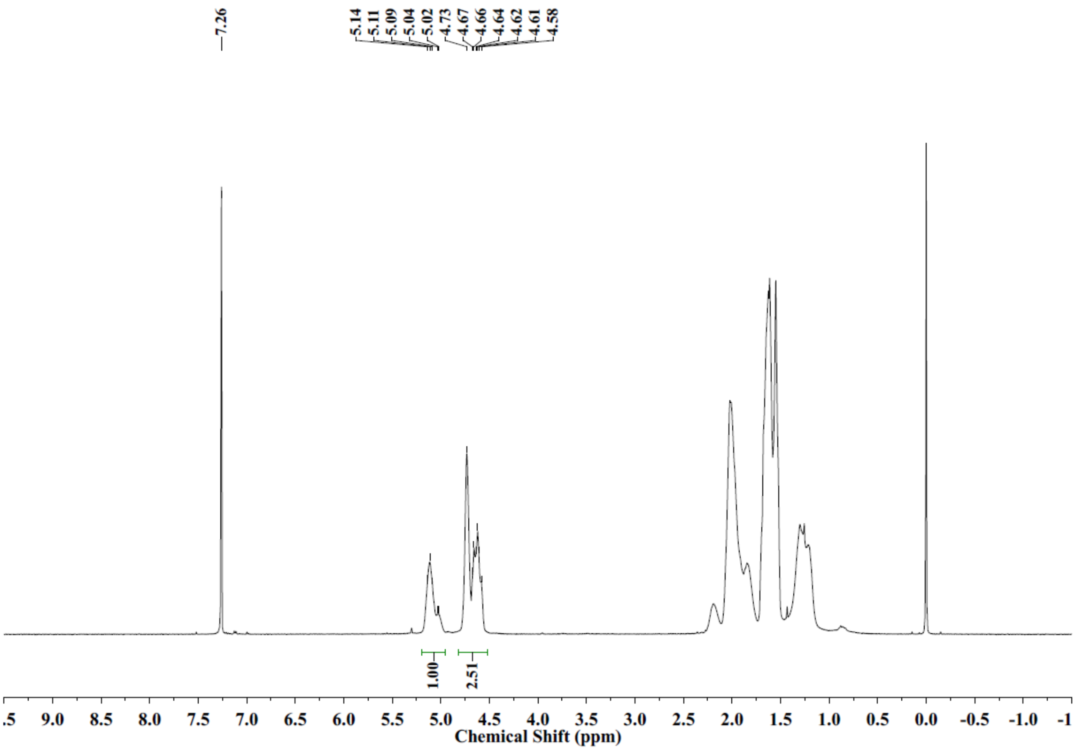

Table 3. entry 1 (^{1}H MMR).

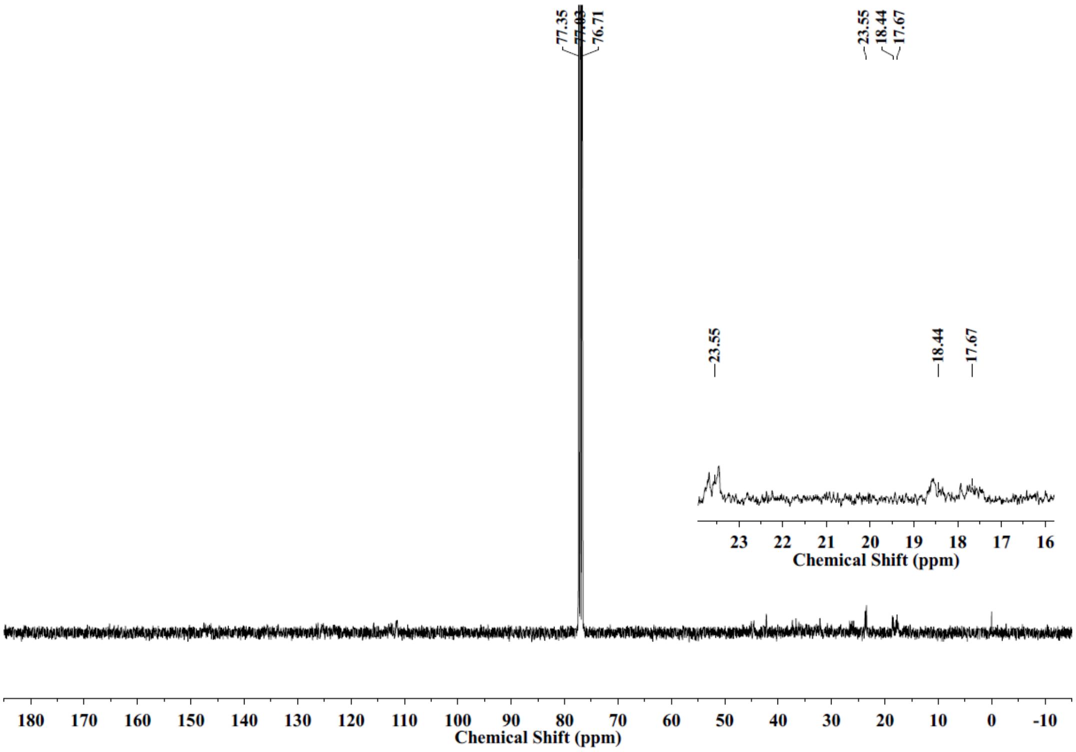

Table 3. entry 1 (^{13}C MMR).

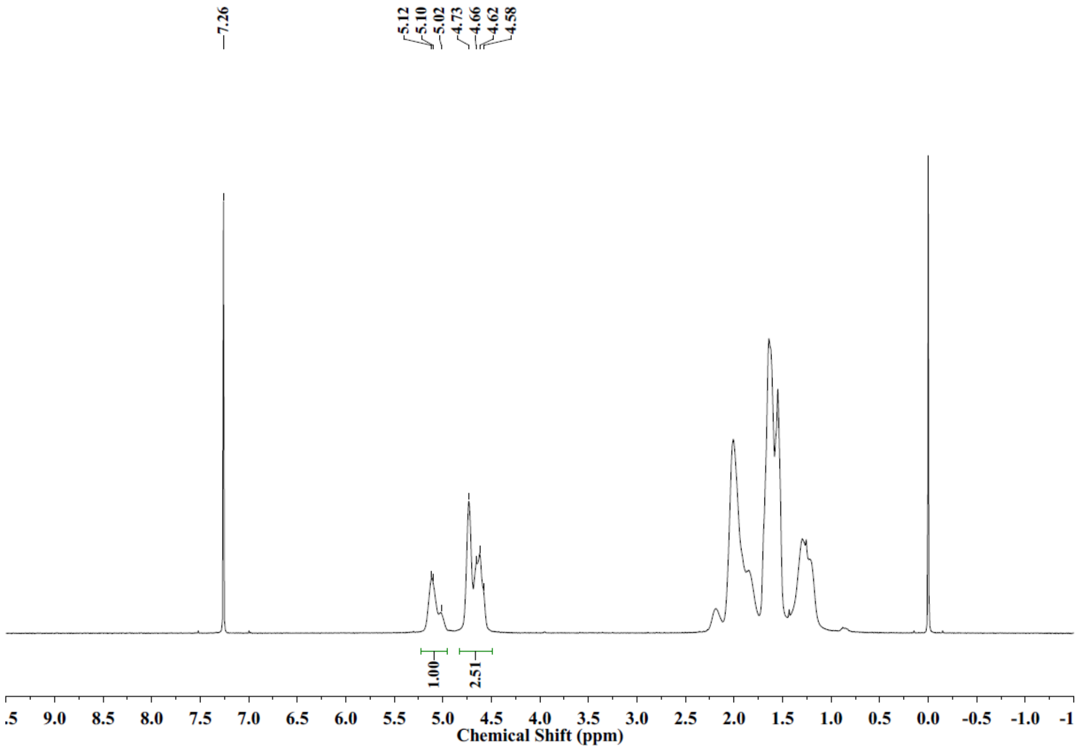

Table 3. entry 2 (^{1}H MMR).

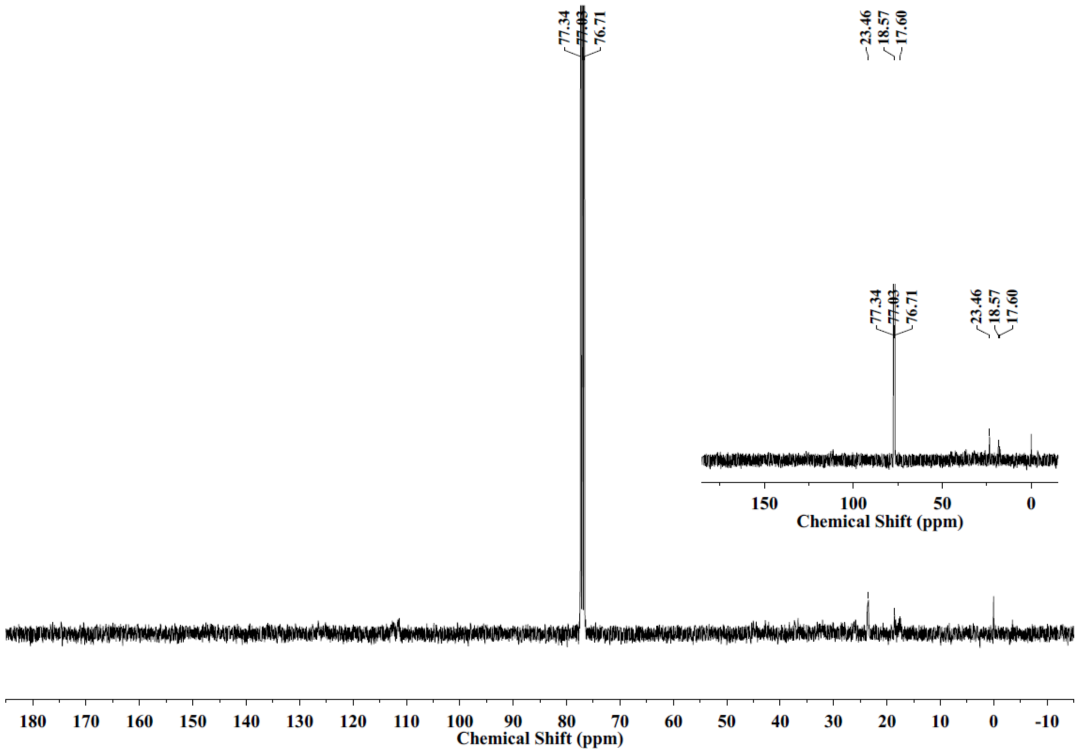

Table 3. entry 2 (^{13}C MMR).

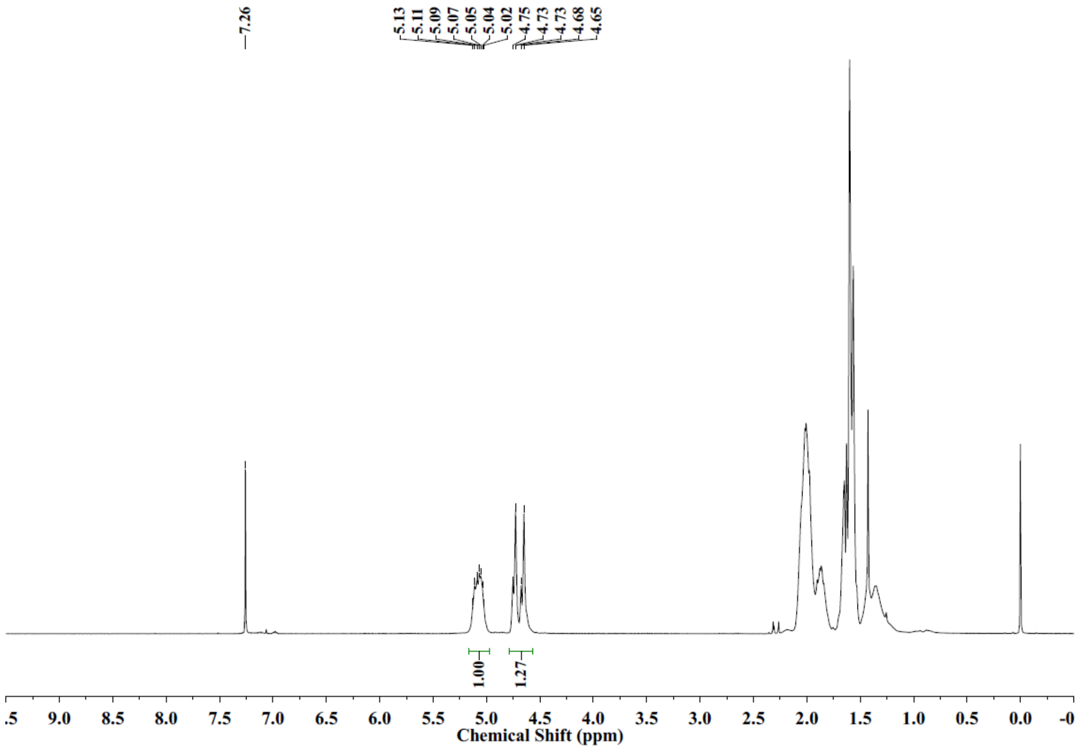

Table 3. entry 4 (^{1}H MMR).

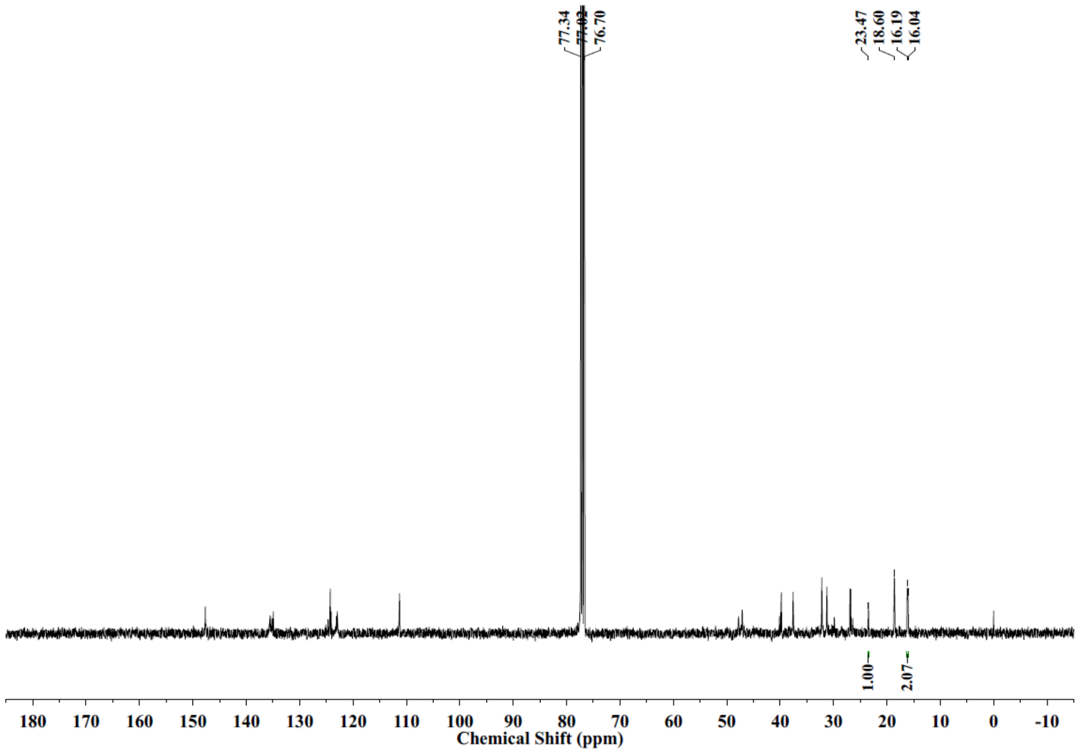

Table 3. entry 4 (^{13}C MMR).

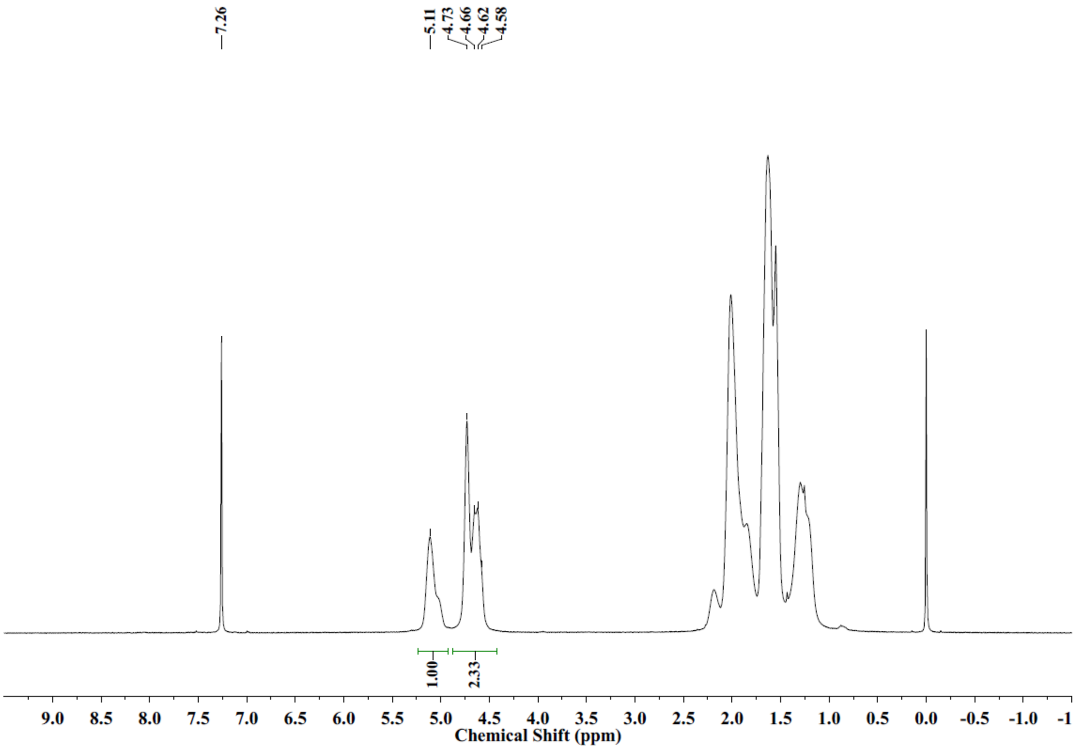

Table 3. entry 5 (^{1}H MMR).

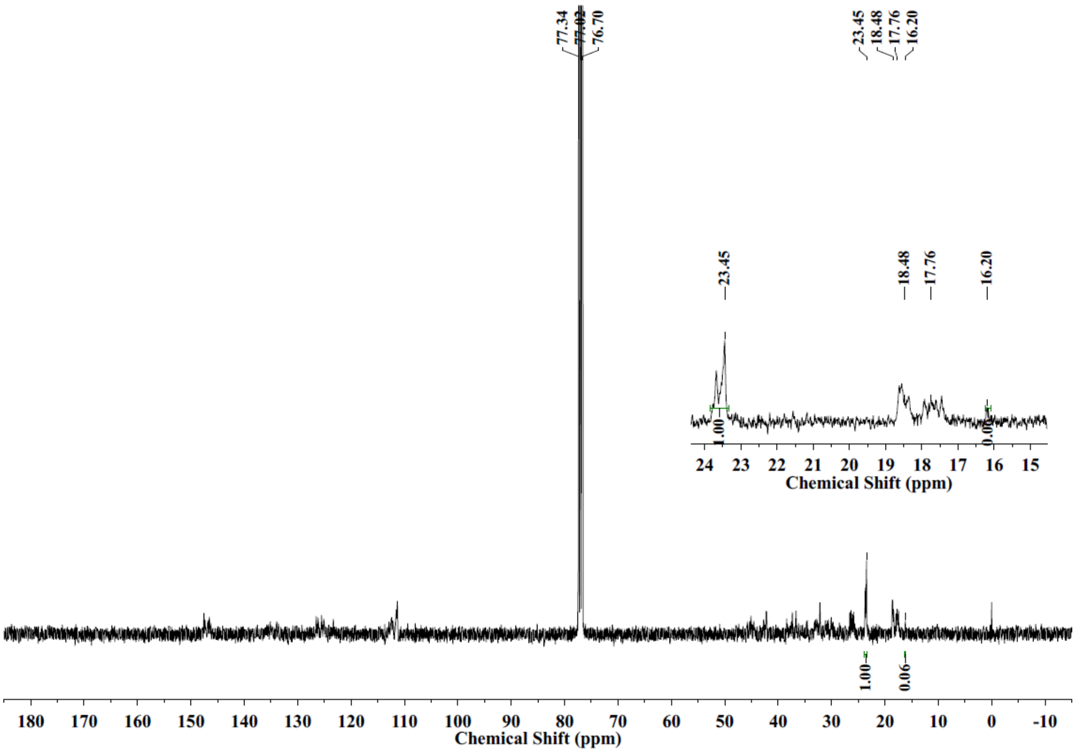

Table 3. entry 5 (^{13}C MMR).

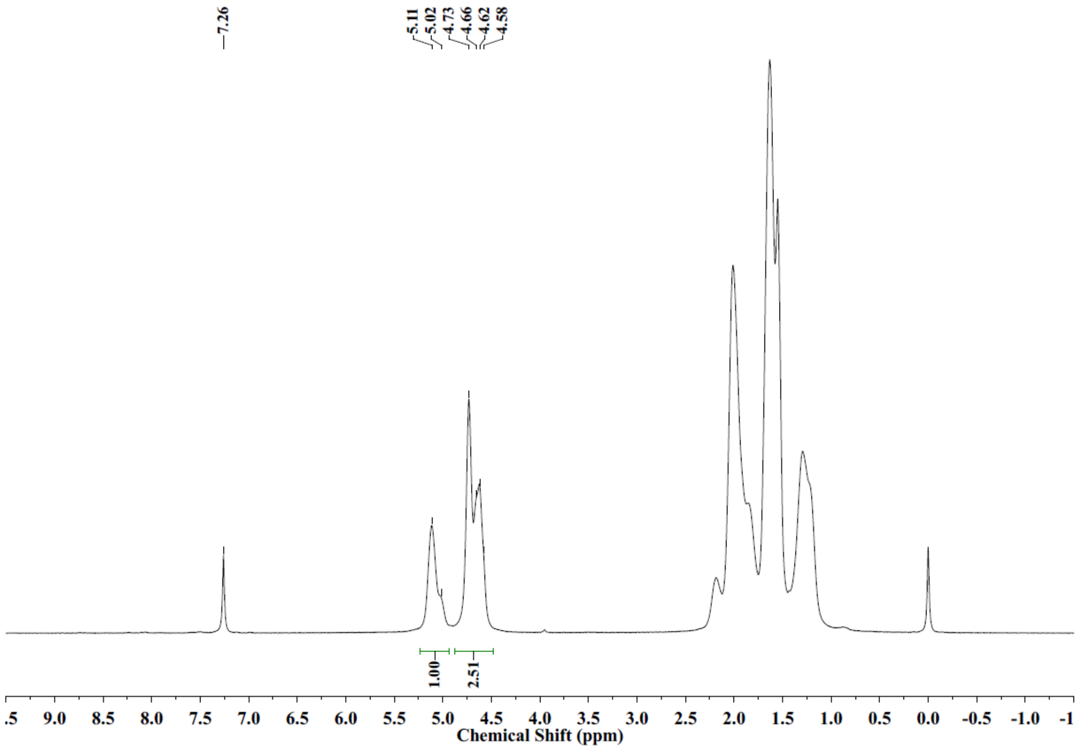

Table 3. entry 6 (^{1}H MMR).

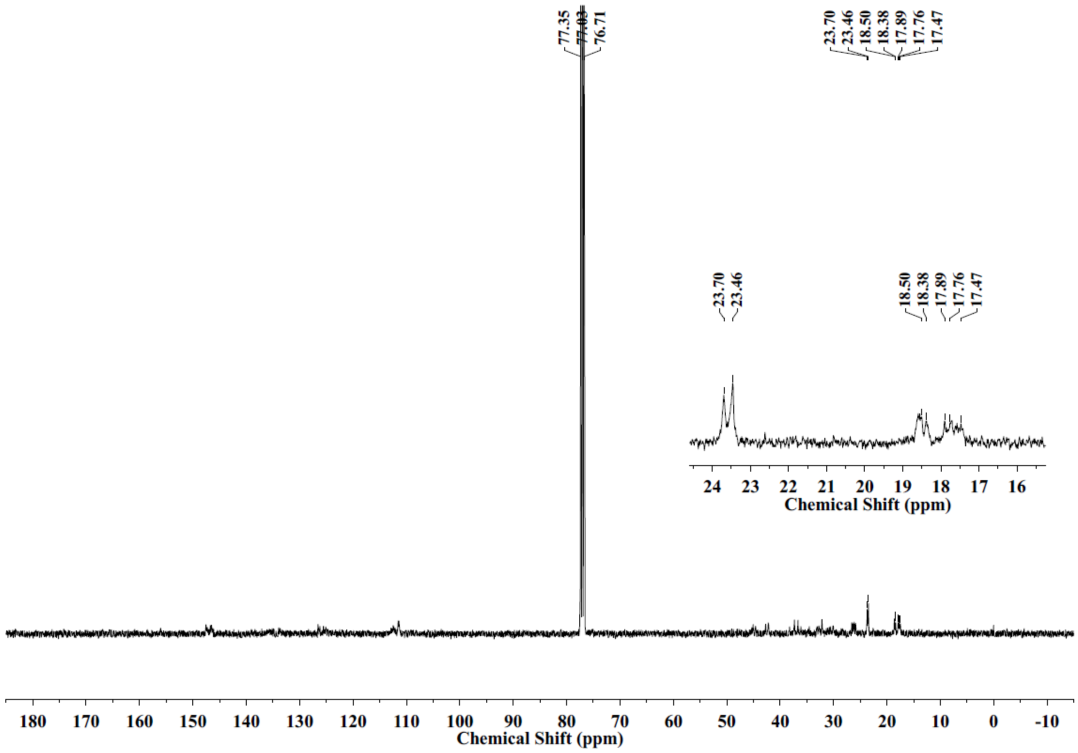

Table 3. entry 6 (^{13}C MMR).

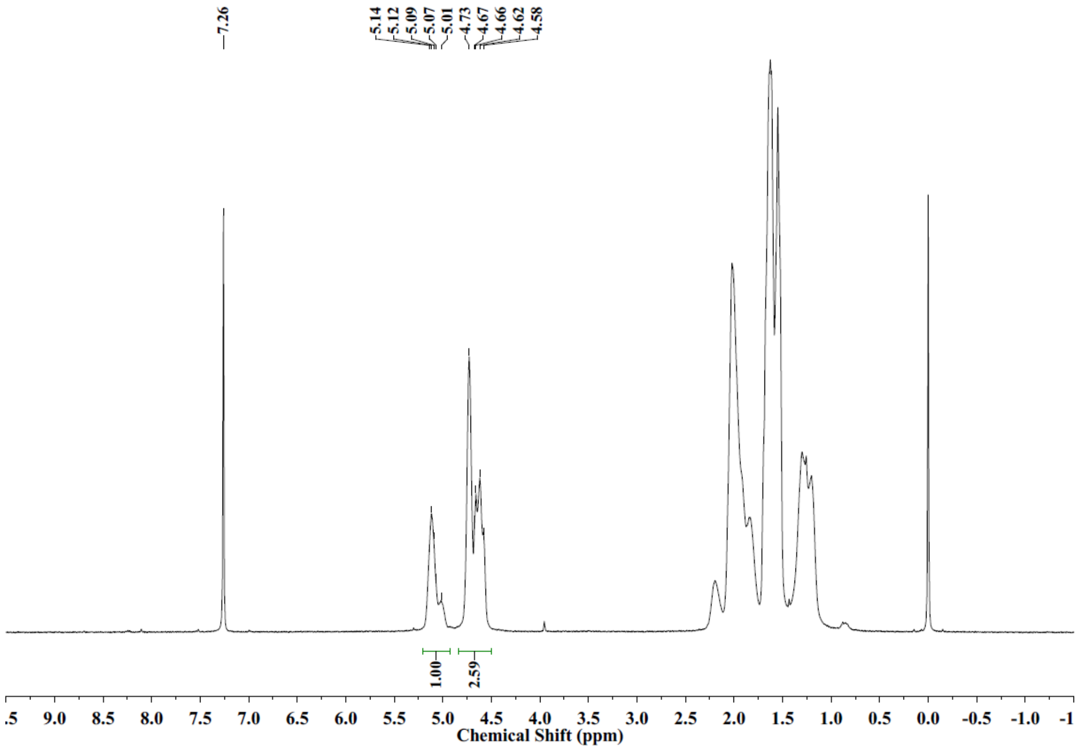

Table 3. entry 7 (^{1}H MMR).

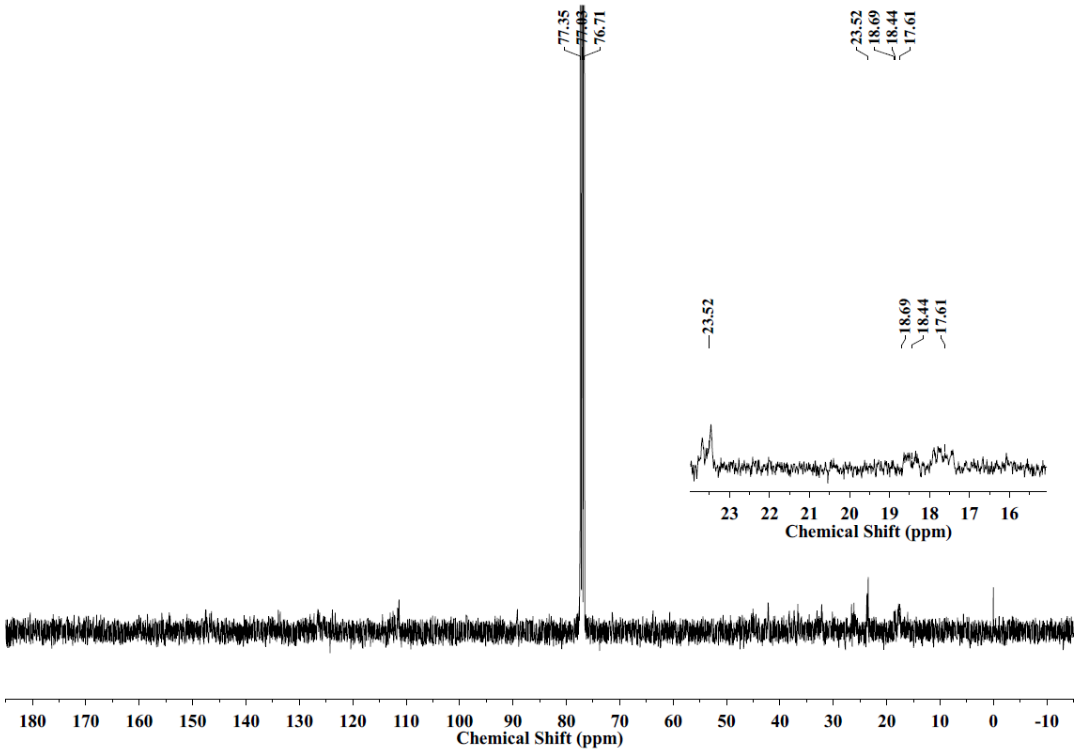

Table 3. entry 7 (^{13}C MMR).

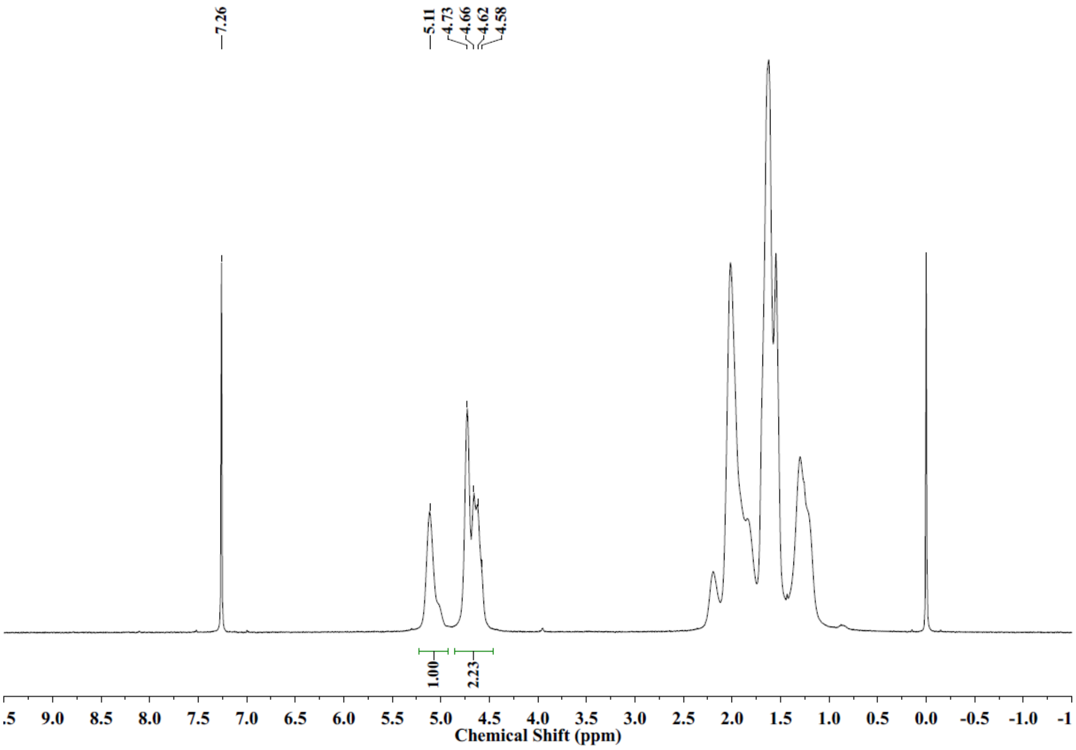

Table 3. entry 8 (^{1}H MMR).

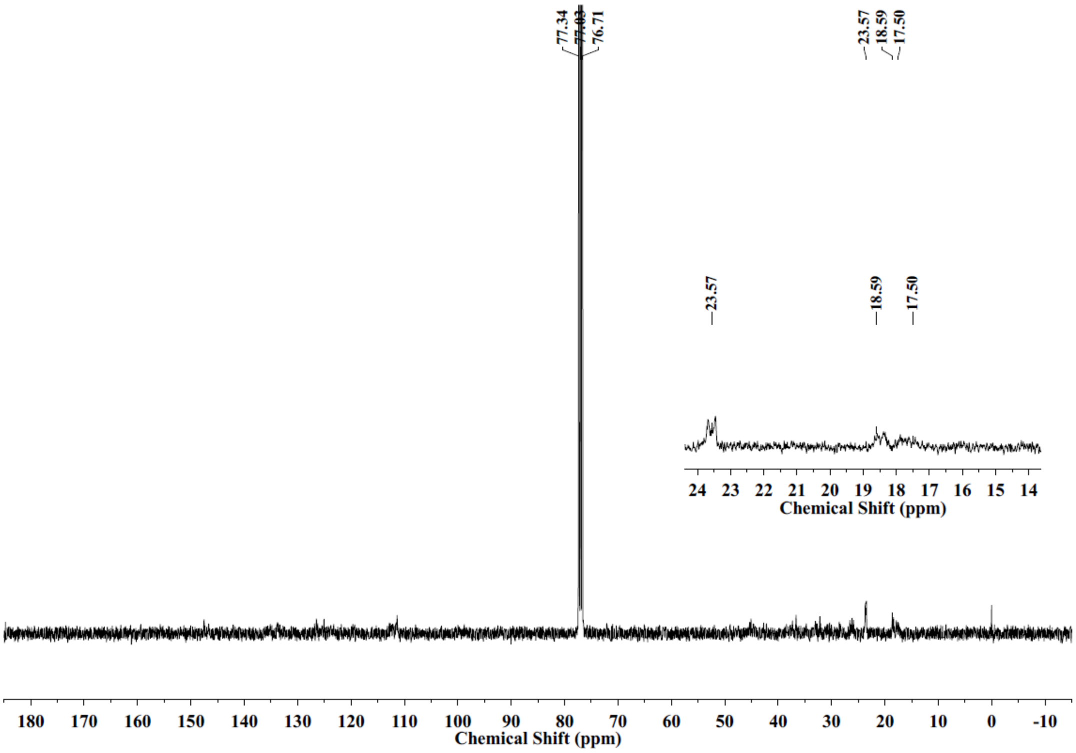

Table 3. entry 8 (^{13}C MMR).

GPC data for polyisoprene

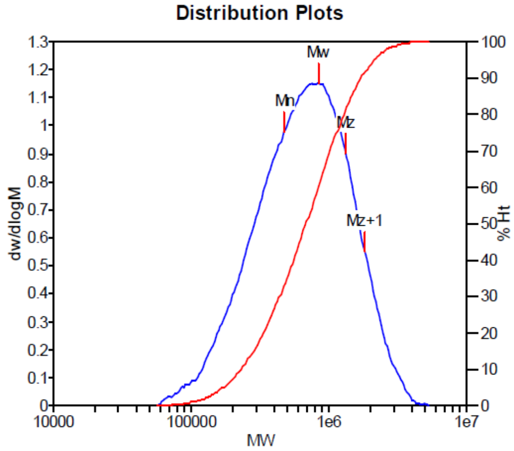

Table 1, entry 1

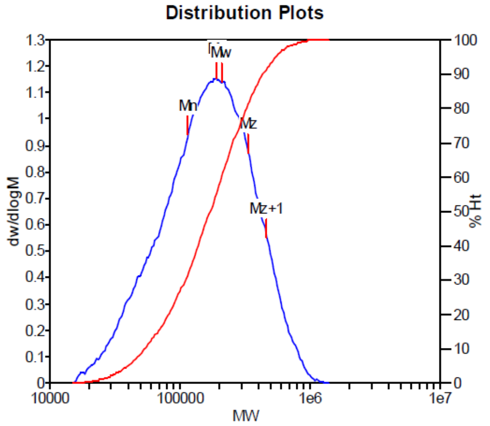

Table 1, entry 2

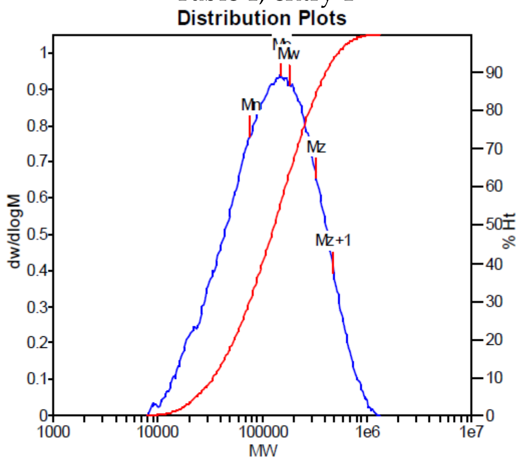

Table 1, entry 3

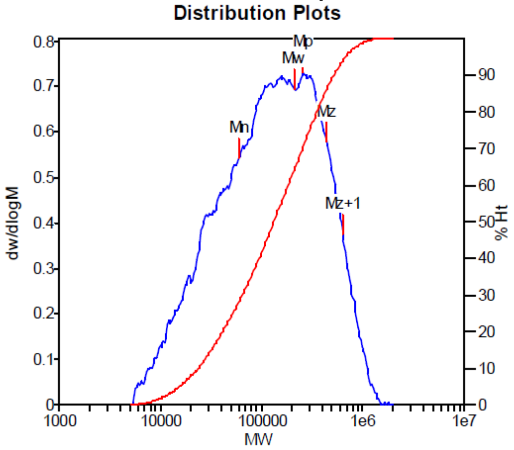

Table 1, entry 4

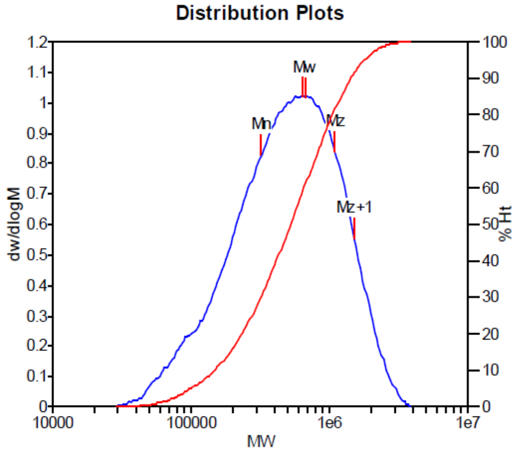

Table 1, entry 5

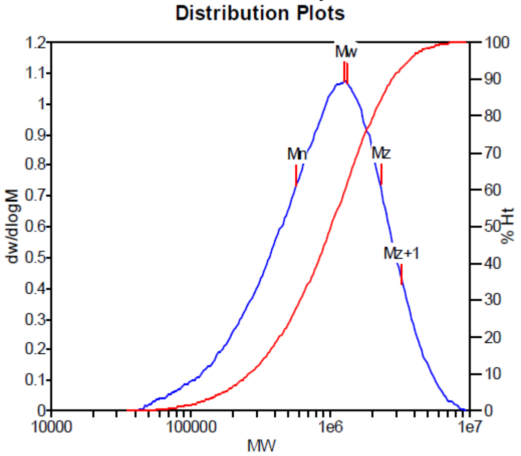

Table 1, entry 6

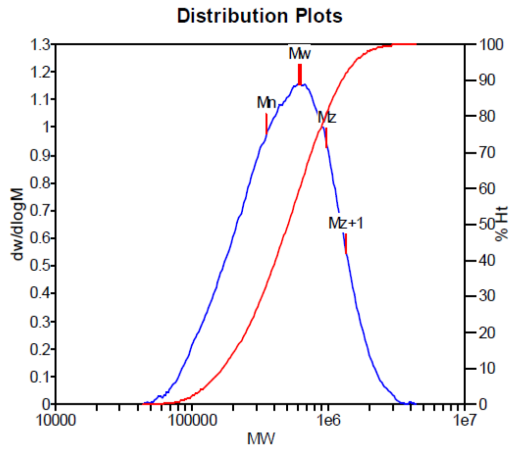

Table 2, entry 1

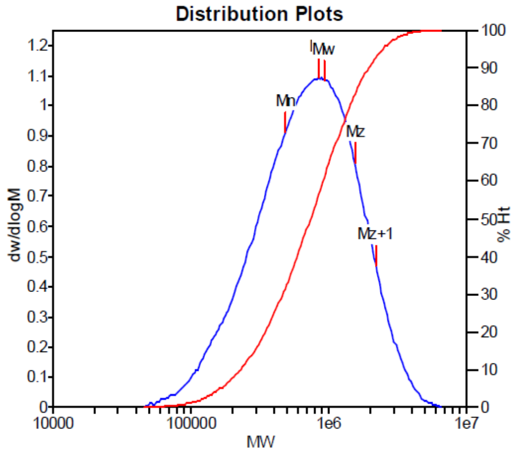

Table 2, entry 2

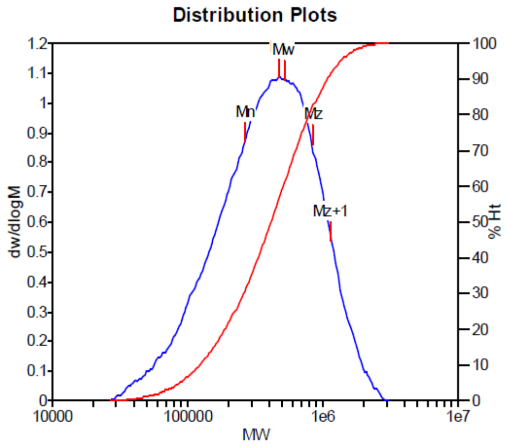

Table 2, entry 3

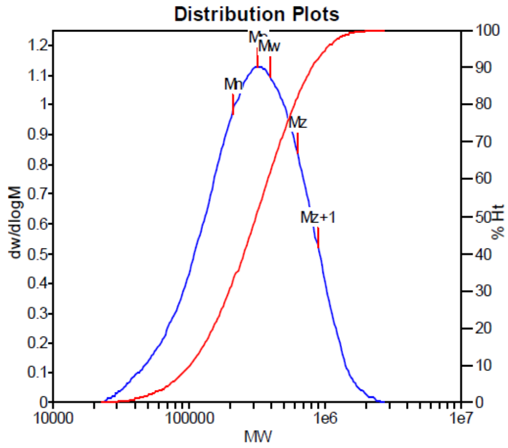

Table 2, entry 4

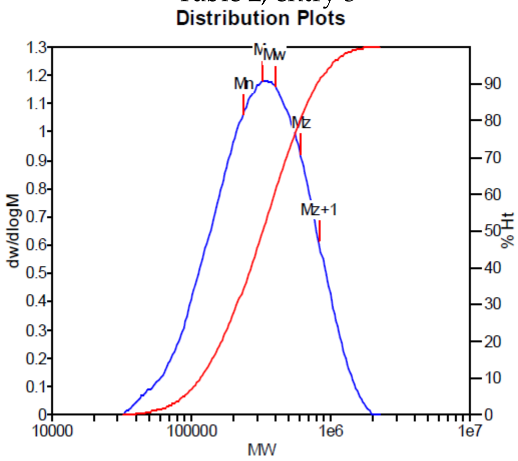

Table 2, entry 5

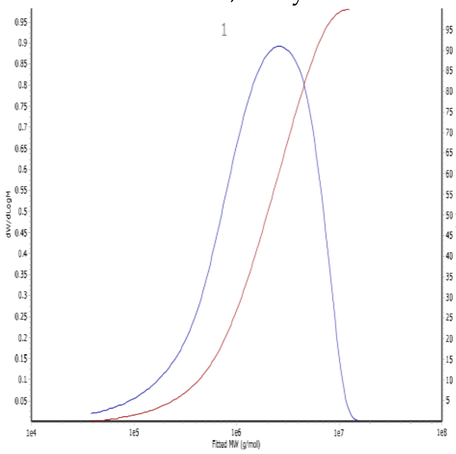

Table 2, entry 6

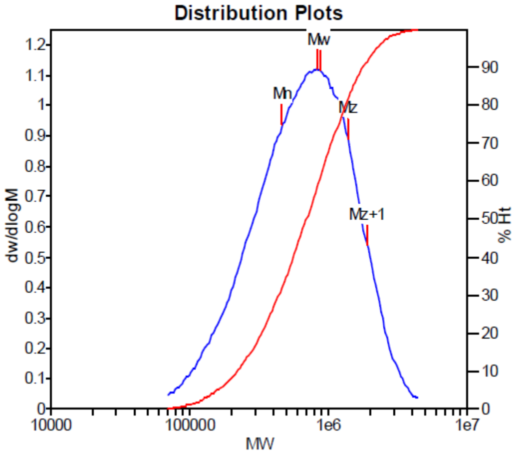

Table 2, entry 7

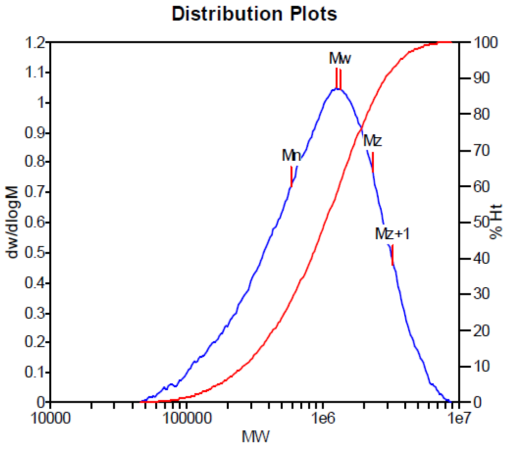

Table 2, entry 8

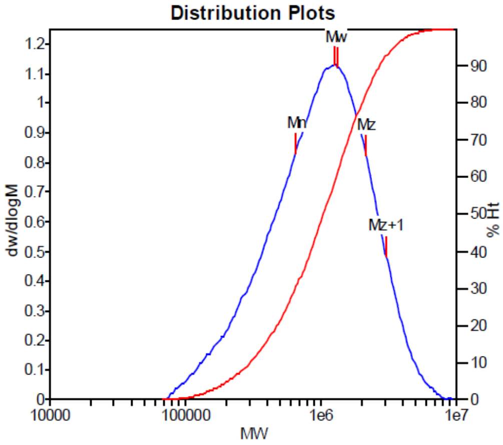

Table 2, entry 9

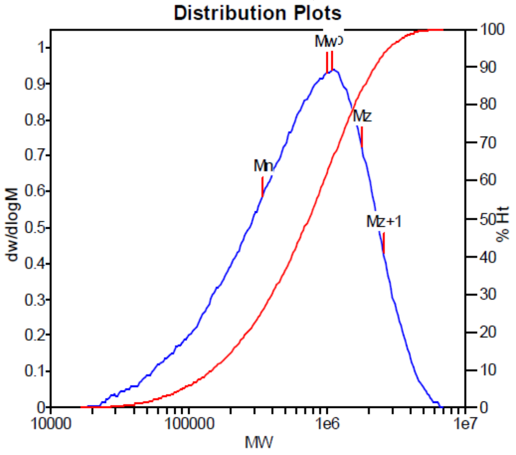

Table 2, entry 10

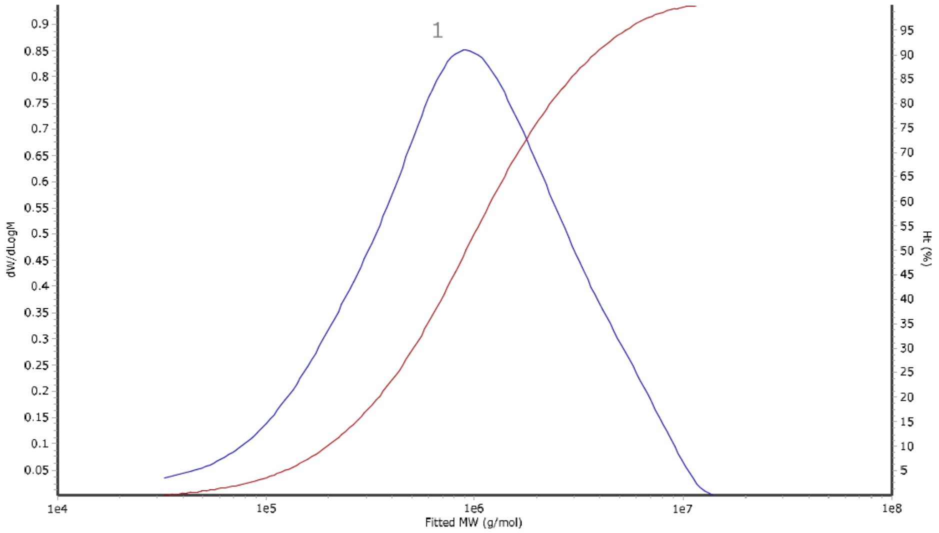

Table 2. entry 11.

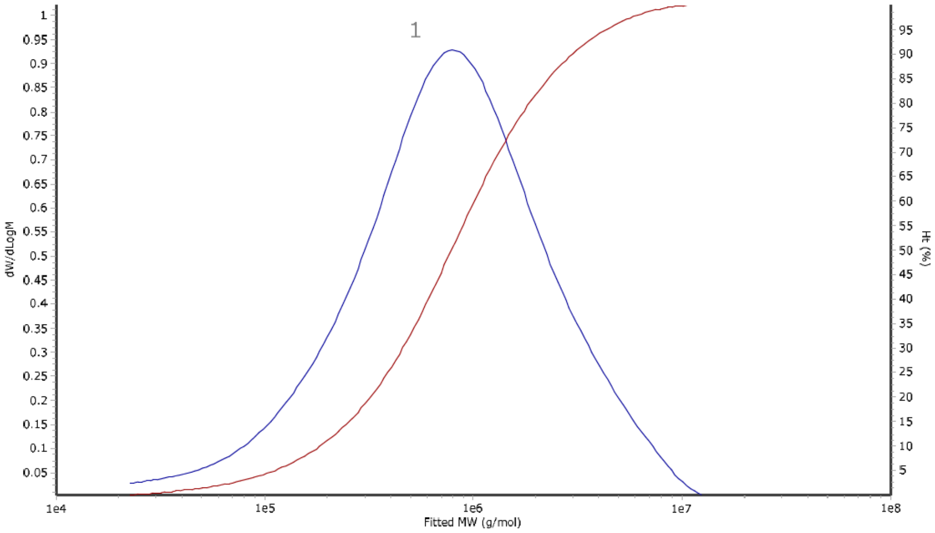

Table 2. entry 12.

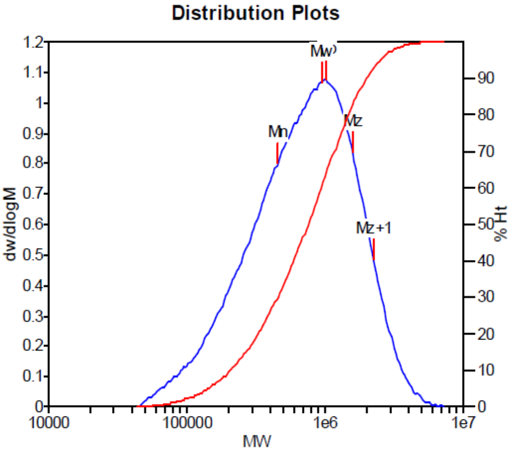

Table 2, entry 13

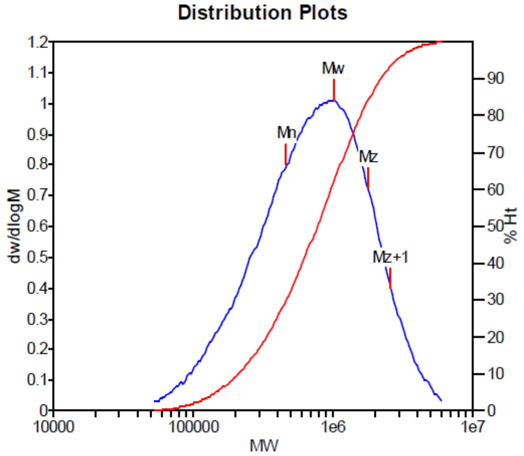

Table 2, entry 14

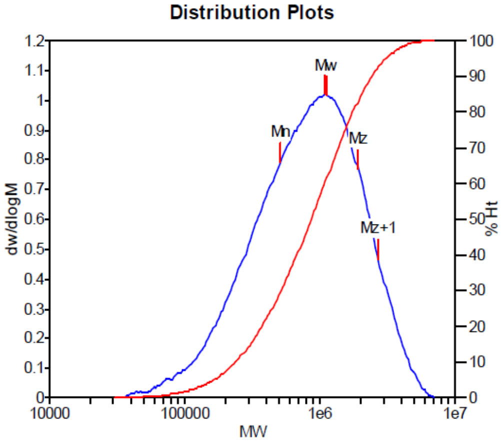

Table 2, entry 15

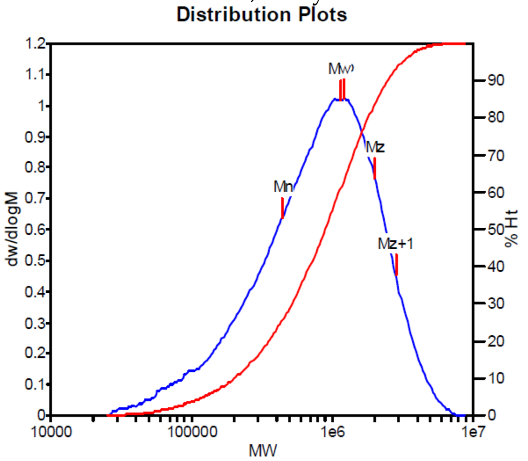

Table 2, entry 16

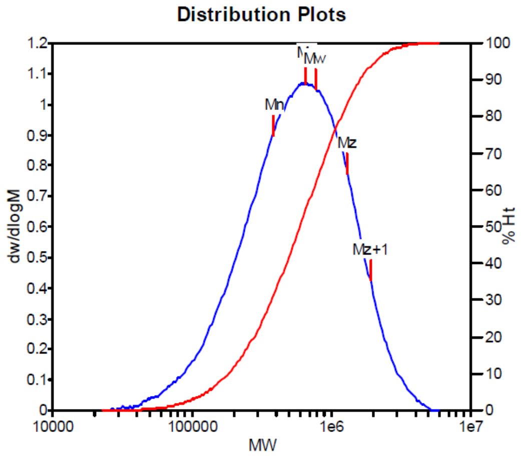

Table 2. entry 17.

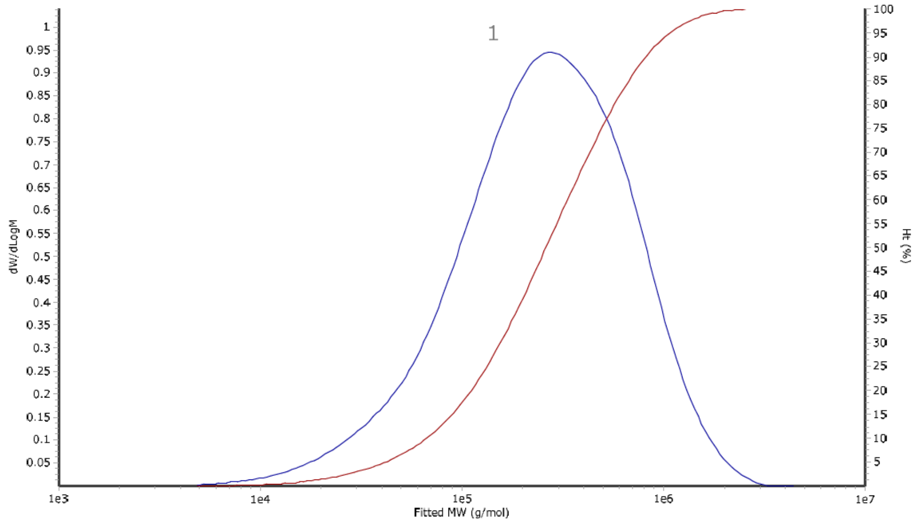

Table 3. entry 1.

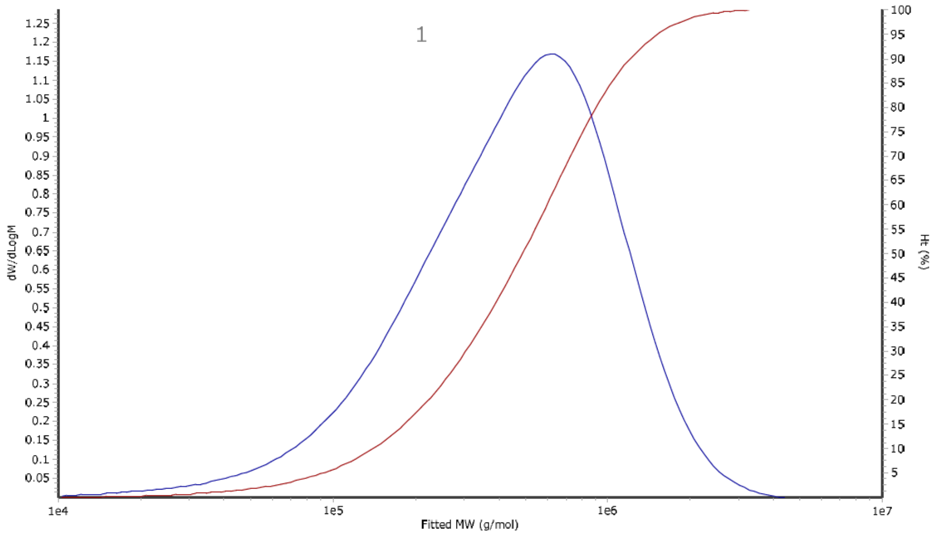

Table 3. entry 2.

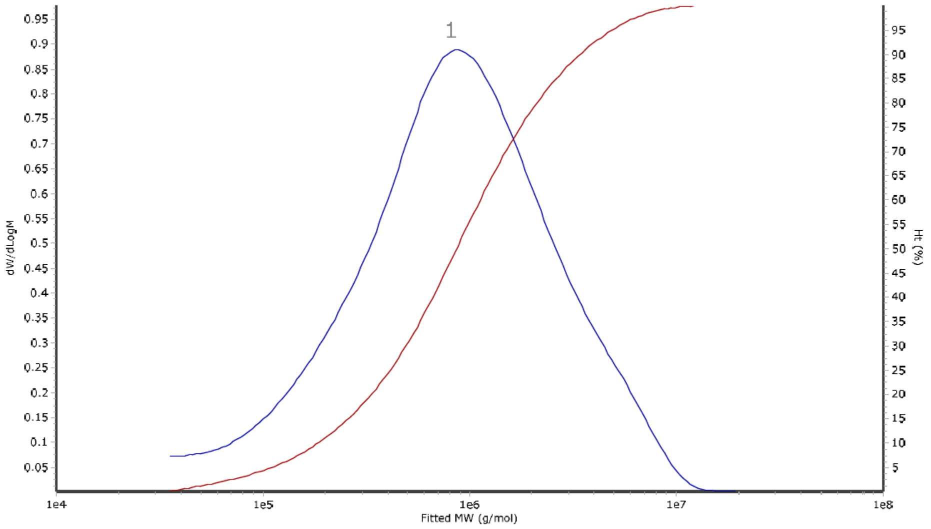

Table 3. entry 4.

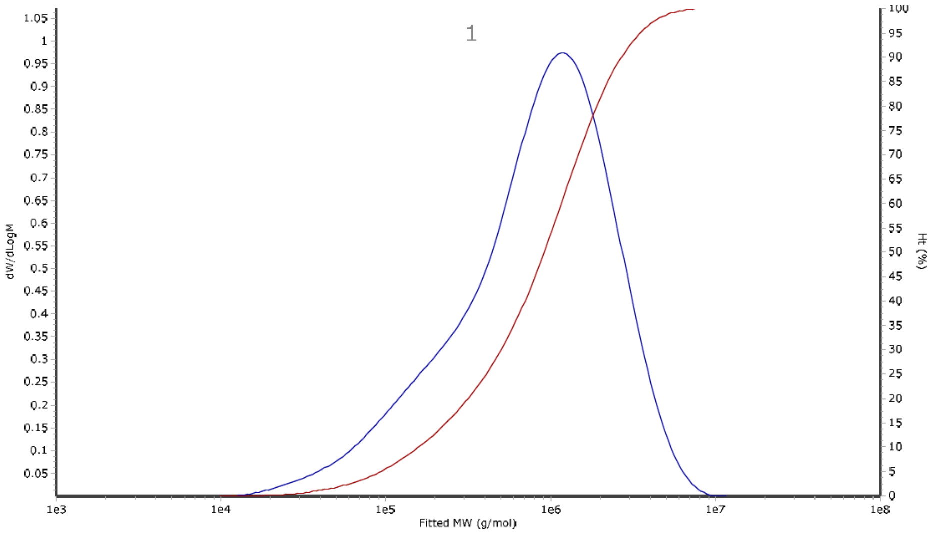

Table 3. entry 5.

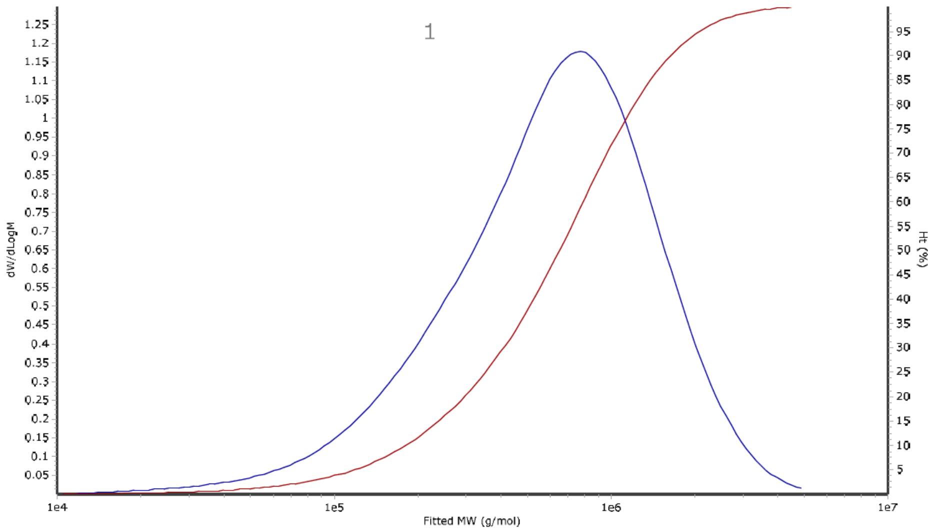

Table 3. entry 6.

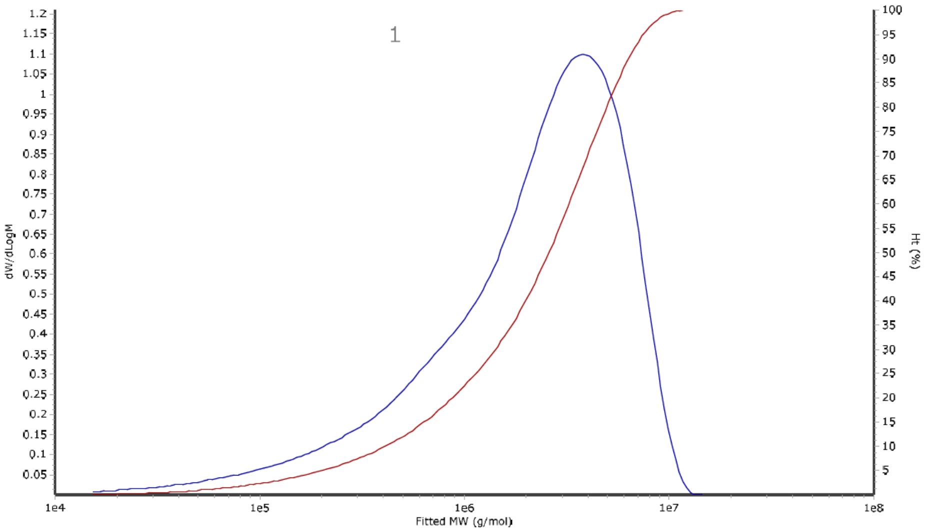

Table 3. entry 7.

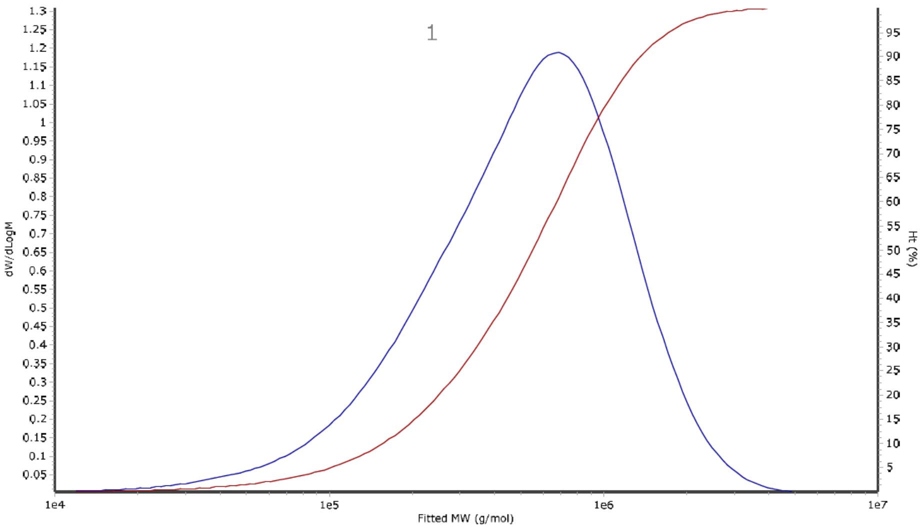

Table 3. entry 8.

Table S4. Comparison of previous works and this work

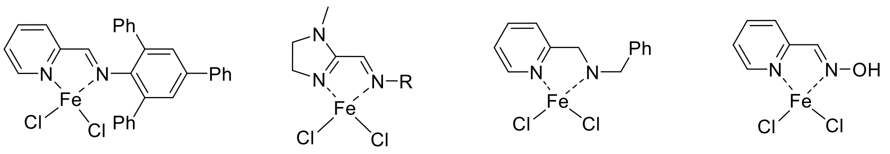

A [ref. 47, 48] B [ref.52] C [ref. 54] **This word**

Catalytic system	Cat.	Time (min)	Yield (%)	Act. (×10⁻⁴)	M_n (×10⁻⁵)	M_w/M_n	Microstructure(%)		
							trans-1,4	*cis*-1,4	3,4
Binary	A	120	98	8.3	10.5	2.0	3	63	34
	B	-	-	-	-	-	-	-	-
	C	10	>99	81.6	4.1	4.0	23	29	48
	D	10	>99	81.6	35.4	1.8	0	47	53
Ternary	A	120	>99	3.7	15.0	1.9	67	0	33
	B	120	88	3.7	0.2	4.4	96	0	4
	C	-	-	-	-	-	-	-	-
	D	2	96	391.7	14.0	2.6	0	44	56
